# Classification of battery compounds using structure-free Mendeleev encodings

**DOI:** 10.1186/s13321-024-00836-x

**Published:** 2024-04-26

**Authors:** Zixin Zhuang, Amanda S. Barnard

**Affiliations:** School of Computing, Australian National University, 145 Science Road, Acton, 2601 ACT Australia

**Keywords:** Machine learning, Encoding, Supervised learning, Classification, Chemical formula, Battery

## Abstract

**Abstract:**

Machine learning is a valuable tool that can accelerate the discovery and design of materials occupying combinatorial chemical spaces. However, the prerequisite need for vast amounts of training data can be prohibitive when significant resources are needed to characterize or simulate candidate structures. Recent results have shown that structure-free encoding of complex materials, based entirely on chemical compositions, can overcome this impediment and perform well in unsupervised learning tasks. In this study, we extend this exploration to supervised classification, and show how structure-free encoding can accurately predict classes of material compounds for battery applications without time consuming measurement of bonding networks, lattices or densities.

**Scientific contribution:**

The comprehensive evaluation of structure-free encodings of complex materials in classification tasks, including binary and multi-class separation, inclusive of three classifiers based on different logic function, is measured four metrics and learning curves. The encoding is applied to two data sets from computational and experimental sources, and the outcomes visualised using 5 approaches to confirms the suitability and superiority of Mendeleev encoding. These methods are general and accessible using source software, to provide simple, intuitive and interpretable materials informatics outcomes to accelerate materials design.

## Introduction

Classification of materials based on their chemical composition is an important task for various reasons, including quality control [44], safety [25, 37], regulation and compliance [6], and life-cycle management [34]. It is also a valuable tool during development (regardless of the size of the data set [54, 66]), since identifying and assigning the class of a material can aid in design, and the selection of materials that more closely meet the requirements of various industries [26], from aerospace and automotive to electronics and healthcare. In medicine and pharmaceuticals, materials are typically classified based on drug formulation, medical device development, and treatments [12]. The classification helps ensure the safety and efficacy of the final products. In electronics, materials can be classified based on selectivity, sensitivity, specificity or economic viability [7, 16, 31, 40, 53].

Classification also aids in new scientific understanding [39, 62, 67], simplifying the study of materials chemical, physical, and structural characteristics and properties. Machine learning (ML) [3, 8, 28, 30, 56, 59] offers a variety of approaches to classification, predicting the classes of materials (the labels) based on the physicochemical characteristics (the features). During discovery and design the possible categories are not always known, and unsupervised learning can be used to identify hidden patterns, trends and relationships among different materials based on their similarities in a high-dimensional feature space, regardless of their functional properties [4, 9, 22, 24, 47, 55, 61]. Coupling clustering with classification can be a useful way to find new classes of materials that are difficult to identify based on intuition[48–50, 67]. In most cases, however, a lot of information about the materials chemistry is required to train the models, making it difficult to focus the research on specific classes with promising applications without committing a lot of time and money to structural characterisation.

Recently a comprehensive assessment of structure-free encodings of complex material was reported, using only the information from the chemical formula [69]. This paper evaluated three structure-free encodings with increasing information content (one-hot, Mendeleev and Mendeleev+ encoding), using three data sets of complex materials used for battery applications and four different unsupervised learning methods, inclusive of six algorithms with four evaluation metrics, in addition visualisations of the results. Although unsupervised learning lacks a ground truth, it is less susceptible to p-hacking by using only the feature space differences, which could be entirely attributed to the different encodings. The encodings compared are available in commonly used informatics platforms [1, 15, 35, 63–65], but it was unclear from this study if similar performance could be expected in supervised tasks, and since the unsupervised methods were uninterpretable, what useful insights can be obtained from models that are ignorant of bonding, symmetry, density or disorder. Successful and interpretable structure-free models would be invaluable to cheminformatics.

In this study we address these questions, comparing the effectiveness of one-hot, Mendeleev and a Mendeleev+ encoding in classification tasks. To ensure the results are consistent and directly comparable with the unsupervised assessment, we use the same computational and experimental data sets, and the clusters identified by the (superior) agglomerative clustering as the categorical labels. We include three linear and non-linear classification algorithms and three evaluation metrics, across binary and multiple classifications, and interpret the results to provide chemical insights. As we will show, Mendeleev encoding, which is based entirely on elemental compositions, provides consistently accurate and stable results, and maximizes interpretability for both computational and experimental data sets. The inclusion of additional features based on summary statistics in Mendeleev+ encoding results in a marginal improvement in accuracy and a reduction in actionable insights that must be weighed against the simplification of model architectures.

## Methods

The objective of supervised learning [57] is to make a prediction of a target label when information on the physicochemical features of each instance (individual material) is available. Common supervised learning tasks include classification [32, 41] and regression [60]. Classification involves the separation of data instances based on their similarities or differences in a high dimensional space using their features. A classifier is trained (using input training data) to recognize how unseen instances relate to some known categories of known instances and assigns them accordingly. There are numerous classification algorithms available, and the superiority of one over another depends on the application and the data set. In this study, three classification algorithms were chosen, and the scikit-learn [51] implementation was used for all. We include logistic regression, as it is provides an interpretable model using a linear decision boundary and probabilistic logic, and is conventionally used as a baseline classifier; decision trees as a non-linear example of an interpretable tree-based model, which uses recursive partitioning logic; and support vector machines based on margin maximization to select the hyperplane that best separates the classes in the feature space, using geometric margin maximization logic.

### Logistic regression (LR)

LR is a simple algorithm that finds a linear correlation among features by fitting a linear regressor model to the feature space, with the feature space preferably linearly separable. The output is then converted to a probability value through a logistic function, known as a sigmoid function:

1 $$\begin{aligned} \phi (z) = \frac{1}{1 + e^{-z}} \end{aligned}$$

where, ${z = {\textbf {w}}^{T}{} {\textbf {x}}}$ is the raw output. The loss function used to optimize the model is the cross-entropy loss. To extract meaningful feature importance from this model, the input vector **x** should be scaled prior to training, such that all features are on a similar numerical scale. The scaling method used to achieve this can be found in the Encoding section. Feature importances are simply the optimized weight vector **w**.

LR is known to perform very well on binary or multiple linearly separable classes, and the importance of features can be clearly reflected by the magnitude of the corresponding coefficients (weights) [14]. This method was included here to explore whether there is a linear relationship between features and class labels previously obtained by agglomerative clustering, since the other classifiers (described below) do not discriminate between linear and non-linear relationships. Logistic regression therefore provides a baseline.

### Decision trees (DT)

DT classifiers are non-linear, non-parametric models based on simple decision rules inferred from the structural features, and trained by recursively splitting the data to predict binary and multi-class problems. They are simple to understand, and an explanation for the condition can be easily obtained using boolean logic. Advantages of DTs are that they require little data preparation and they can be validated using statistical tests. Disadvantages include possible instability with respect to small variation in the data, locally optimal decision at nodes dominate since they are based on heuristic algorithms (given that an optimal decision tree is known to be NP-complete), biased trees can be created if some classes dominate, and they are prone to over-fitting [10].

### Support vector machine (SVM)

SVM seeks the optimal partitioning hyperplane to split sets of vectors and generate support vectors on either side of the hyperplane (i.e. the vectors with the shortest geometric distance from the hyperplane). SVMs are characterized by sparse solutions and Vapnik-Chervonenkis control of the margin and the number of support vectors, resulting in an effective tool in real-value function estimation. Fundamentally, SVMs are linear classifiers and require non-linearly separable problems to be converted into linearly separable problems using the kernel trick. This is achieved by mapping non-linearly separable data into a higher dimensional space [11] via a mapping function. In the present study we included the selection of the kernel as a hyper-parameter to be tuned during optimization, as the selection of the appropriate kernel is non-trivial.

SVM was chosen as it is known to generalize well on unseen data, and the computational complexity does not depend on the dimensionality of the input space. SVMs are good at avoiding local minima and generally exhibit less over-fitting than other sophisticated algorithms [52]. SVMs require careful tuning as model performance is sensitive to the hyper-parameters. In the present study, we optimized the regularization parameter, the tolerance for stopping criterion, the maximum iterations for the solver, and a number of parameters related to the kernel such as the kernel coefficient, the independent term and true degree of the polynomial (should that kernel be selected).

### Encodings

The simplest machine readable format for chemical formulas can be is *one-hot encoding*. This encoding expands the single categorical information into a $1\times N$ matrix (vector) with entries that are either 0 or 1. All material compounds can be encoded using one-hot encoding, but it does not encode stoichiometric differences.

Another simple, modified structure-free encoding is *Mendeleev* encoding [69], which is chemically intuitive and is capable of discriminating between compounds that would share the same one-hot matrix. In Mendeleev encoding the categorical compounds are expanded into a $1\times N$ matrix (vector) with entries that reflect the chemical composition. This encoding can accommodate non-stoichiometric chemical formulae (provided *x* is assigned a value).

A more complex structure-free encoding can include features based entirely on properties of the periodic stable such as atomic weights, group or period. This has been refereed to as *Mendeleev+* encoding, with features including statistics such as the minimum, maximum, range, mean and standard deviation, with properties such as molar volume, density, atomic weight and volume, the atomic number and Mendeleev number, the column, row, block and group of the atom, and the number of valence electrons. These non-integer ‘artificial’ features can make the data instances (compounds) more distinguishable, even though entirely different chemical formulae can give the same feature value [69].

To normalize the feature values, a global Min-Max scaling was applied to all Mendeleev features, such that all values are in [0,1]. For the additional Mendeleev+ features, each feature was individually scaled to have range [0,1].

Previous work has found that Mendeleev encodings are more accurate, stable and reliable than one-hot encoding in unsupervised tasks, and may be more sensitive to the nature of a compound than just the constituent elements (less additive). Principle component analysis of Mendeleev encoded materials captured more of the variance in fewer components than one-hot encoded materials, as did archetypal analysis to reduce the instance space. Mendeleev encodings consistently resulted in superior clustering outcomes, as determined by the silhouette, Calinski-Harabasz and Davies-Bouldin scores.

### Evaluation

In this study, the results from each classifier have also been evaluated using a number of techniques and metrics. To begin with, learning curves were generated for each training process to determine a number of factors. The training curve is used to identify any bias error leading to under-fitting, which occurs when the model is not sophisticated enough to effectively fit the data. This indicates that the model cannot sufficiently describe the trends in the data. The *k*-fold cross-validation curves are used to identify any variance error leading to over-fitting, which occurs when the model is too sophisticated and effectively fits to the noise. This indicates that the model cannot generalize to unseen data. We look for convergence with training and cross-validation curve to identify whether performance could be improved with the addition of more training data, or if sufficient data has been provided for the choice of model and hyper-parameters.

The performance of the classifiers on unseen data in the testing set is evaluated using classification reports, based on the number of true positives (TP), true negatives (TN), false positives (FP) and false negatives (FN). The reports include the precision (positive predictive value, TP/[TP+FP]) and the recall (sensitivity, TP/[TP+FN]). Accuracy (measured here using the F1-score) is simply a ratio of correctly predicted observation to the total observations, such that: F1-score = 2 $\times$ ([precision $\times$ recall]/[precision+ recall]). In addition to classification reports, we also present the fractional number of TP, TN, FP, FN as a confusion matrix, cross referencing the prediction with the ground truth label.

Receiver operating characteristic (ROC) curves are widely used in health and medical informatics [19, 33] to evaluate the sensitivity of classification tasks. ROC curves use the number of FP and TP classifications as the horizontal and vertical axes respectively, and the area under the curve (AUC) measures the successful classification TP and TN rates of the ground truth classes. In this study we provide the AUC-ROC curves in Additional file 1.

In some cases the outputs from the classifiers are interpretable, via feature importance profiles. Both logistic regression and decision trees output a ranked order of the features that reflects how important they are to the architecture of the model, and therefore how important they are to the prediction. They are model specific, and available for both binary and multi-class classification. In the case of decision trees the profiles are determined by how may times the feature is used to split the data at nodes. In the case of logistic regression, the profiles are the weights on each feature and so multiple profiles are generated for each class and these can be averaged to obtain the overall ranking for the multi-class task. In addition to this, we report the architecture of the decision trees.

## Results

Structure-free classification is particularly useful to the discovery and design of materials for energy storage systems such as batteries, due to the large combinatorial space. Batteries are complex electrochemical reaction systems [5, 13, 20] and Li-ion batteries are well established as the benchmark for high energy and power density, and high efficiency and recharging cycles [38, 43, 45]. New high-capacity battery materials [27, 29] that reduce our dependence on Li [42] are an area of intense research, motivated by our need to make the energy economy more sustainable [2, 58]. Research into alternative materials, such as sodium-ion batteries [21, 46] shows promise, and ML has been instrumental in predicting the electrochemical potential for new cathode materials and establishing quantitative molecular structure-redox potentials relationships [17, 36].

In this study, we have used two battery materials data sets to compare the performance of structure-free classification of highly complex chemical systems. Each set represents compounds proposed or currently used for energy storage applications, with different sample sizes, dimensionality and sources. As mentioned above, the categories used as target labels were previously identified using unsupervised agglomerative clustering, as reported in Ref. [69]. Each dataset was split into a training and test set with 85% and 15% of the total data population respectively. All data pre-processing and training was done purely on training set, with only the final testing carried out on the test set. A hyper-parameter optimization searching 20 samples in the LR, DT and SVM training was performed to ensure stability and optimal performance. During optimization we used 7-fold stratified cross-validation, and 10-fold stratified cross-validation were used for computing the learning curve.

### Computational predictions

To explore the utility of the three structure-free encodings in classification tasks, we have used a computational data set of 10,129 instances of battery compounds that was retrieved from the Materials Project online database [23, 68]. The data set was obtained using both the legacy and the new APIs offered by the Materials Project. Each data compounds contributes at least one “voltage pair”, containing the charge and discharge formulas, and the working ion of a single electrochemical reaction step. Post-processing has been applied to remove redundant metadata, and the discharge formula is chosen as the most representative formula of a data instance, since it provides insights into both the working ion and the charge formula. Most battery compounds contain one voltage pair each, but some contribute several voltage steps to the database, and each voltage pair represents a unique data instance.

**Fig. 1:**
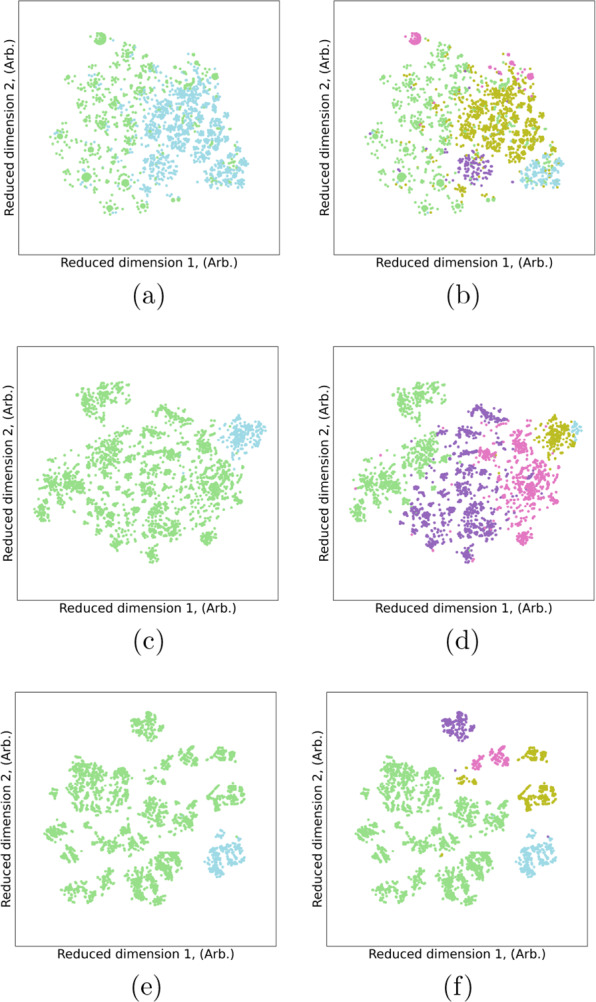
Visualisation the 2-cluster (**a**, **c**, **e**) and 5-cluster (**b**, **d**, **f**) results from agglomerative clustering of battery compounds in the computational data set from Ref. [69], encoded using (**a**, **b**) one-hot encoding, (**c**, **d**) Mendeleev encoding, and (**e**, **f**) Mendeleev+ encoding

The results or the two-cluster and five-cluster cases based on agglomerative clustering from Reference [69] are shown in Fig. 1 for one-hot, Mendeleev and Mendeleev+ encodings, respectively. These results will be used as target labels for binary classification and multi-class classification of this data set. Since the clustering results are uninterpretable, the LR and DT classifiers provide insights into the characteristics of the materials that determine the hidden categories.

#### Binary classification

The results of the binary classification of the computational data set encoded with one-hot, Mendeleev and Mendeleev+ encoding are captured in the classification report in Table 1. It can be observed that all of these structure-free encodings are capable of separating the materials into the unsupervised categories, proving in this case they are separable classes. This is confirmed by the AUC-ROC curves in Additional file 1. Due to the class imbalance, all models report superior accuracy, precision and recall for Class 1 (the majority class). The scores for each model improve as more information is encoded, with Mendeleev+ encoding delivering perfect results every time, while the one-hot encoding has not converged with 8000 training instances and needs more data for all models to improve. From LR to DT to SVM, the accuracy, precision and recall of each encoding generally increases. In each case the model parameter are included in Additional file 1 for reproducibility.

**Table 1:** Binary classification report for logistics regression (LR), decision trees (DT) and support vector machines (SVM) tested on the computational battery compounds data set, encoded using one-hot, Mendeleev and Mendeleev+ encoding

Algorithm	Encoding	Metric	Class 0	Class 1
		Precision	0.981	0.970
	One-hot	Recall	0.979	0.973
		Accuracy	0.980	0.972
		Precision	0.999	1.000
LR	Mendeleev	Recall	1.000	0.992
		Accuracy	1.000	0.996
		Precision	1.000	1.000
	Mendeleev+	Recall	1.000	1.000
		Accuracy	1.000	1.000
		Precision	0.997	0.992
	One-hot	Recall	0.994	0.995
		Accuracy	0.996	0.994
		Precision	1.000	0.992
DT	Mendeleev	Recall	0.999	1.000
		Accuracy	1.000	0.996
		Precision	1.000	1.000
	Mendeleev+	Recall	1.000	1.000
		Accuracy	1.000	1.000
		Precision	0.997	0.997
	One-hot	Recall	0.998	0.995
		Accuracy	0.998	0.996
		Precision	1.000	1.000
SVM	Mendeleev	Recall	1.000	1.000
		Accuracy	1.000	1.000
		Precision	1.000	1.000
	Mendeleev+	Recall	1.000	1.000
		Accuracy	1.000	1.000

The accuracy is measured using the F1-score

**Fig. 2:**
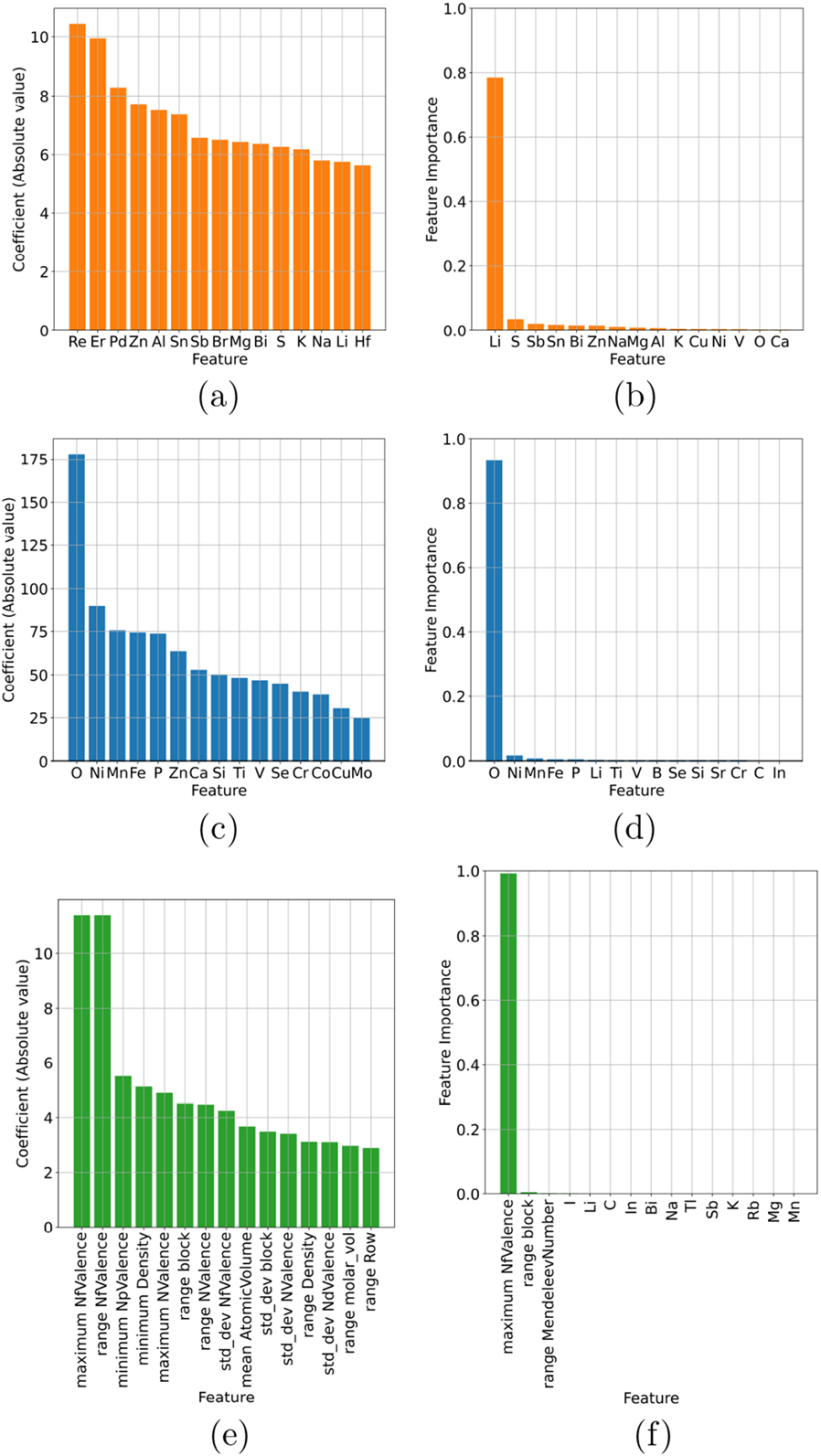
Feature importance profiles showing the top 15 features for binary classification of battery compounds in the computational data set using (**a**, **c**, **e**) logistic regression, and (**b**, **d**, **f**) decision trees, encoded using (**a**, **b**) one-hot, (**c**, **d**) Mendeleev, and (**e**, **f**) Mendeleev+ encoding

**Fig. 3:**
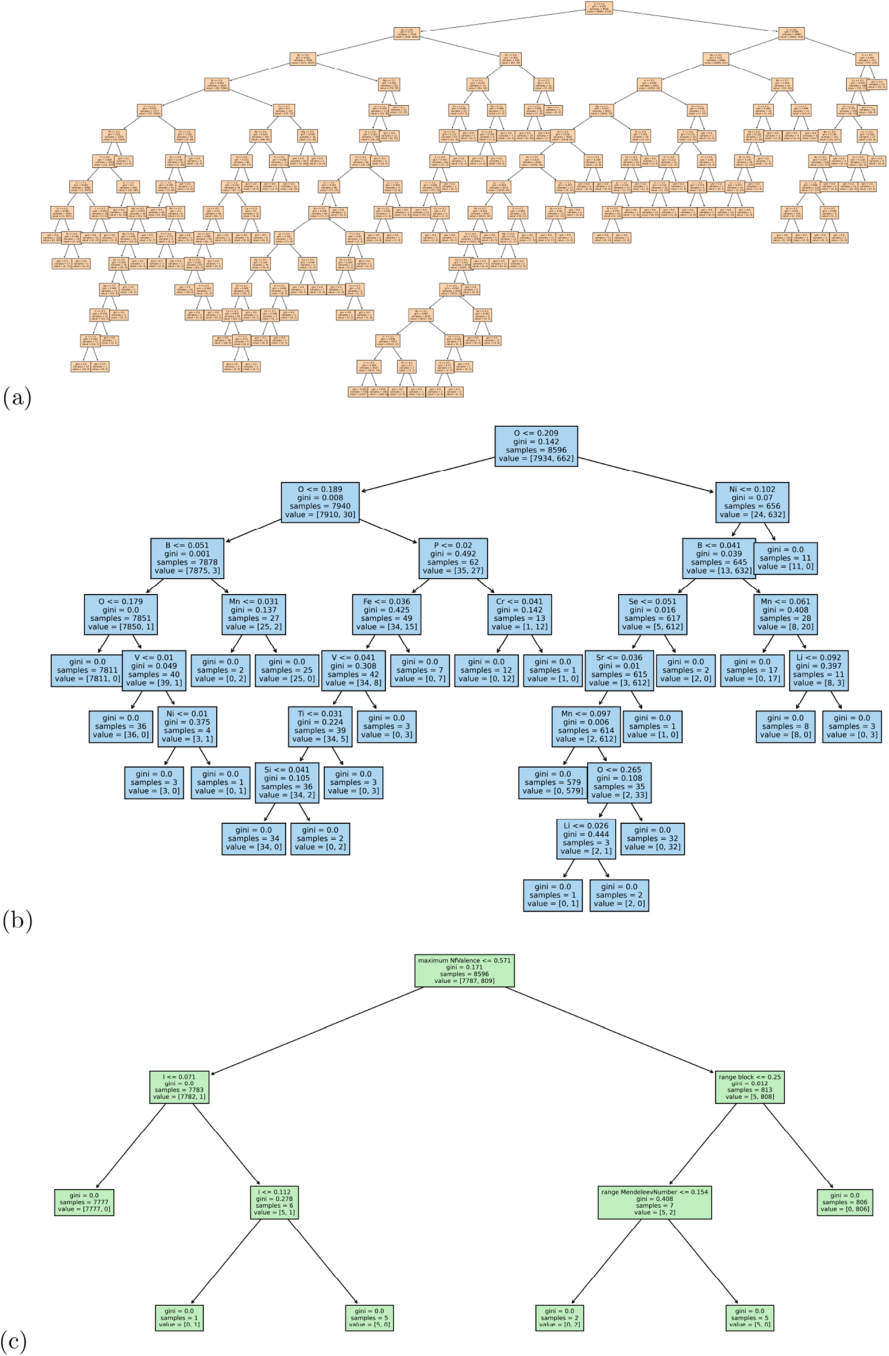
Binary classification decision tree battery compounds in the computational data set, encoded using (**a**) one-hot, (**b**) Mendeleev, and (**c**) Mendeleev+ encoding

The superior results from Mendeleev+ encoding is somewhat diminished when we look for deeper chemical insights. Both LR and DT expose feature rankings that provide insights into the model architecture. These are show in Fig. 2, for the one-hot, Mendeleev and Mendeleev+ encoded data, where we can see that LR treats the feature space more evenly, with a few highly weighted features dominating the models. In contrast, DTs split the data based on one dominant features, and then require deep branches based on the remaining features to separate the materials. Given the high dimensionality of the input data, it is not surprising that the decision trees are deep and complicated. However, as we see from Fig. 3, the complexity of the trees significantly decreases as the encoding includes more information.

The way the structure-free features is used to develop the binary classifiers is consistent across the encodings. The ranking of the important features is less consistent, and less useful. The top 5 one-hot encoded features are entirely different between LR and DT classifiers, and there are only a few elements that are among the top 15 (shown) for both models. The top 5 features for Mendeleev encoded features are the same, regardless of the classifier, and there are other elements consistently appearing in the top 15 ranked features. The top feature for the Mendeleev+ encoded materials is the same for LR and DT, but most other features in the top 15 are different. The top Mendeleev+ encoded features are also quite non-specific, related to things like the maximum number of *f* valence electrons, which can vary a lot between materials, and be the same for different materials. This makes the Mendeleev+ encoded features less interpretable, and the classes less useful for applications or further design.

#### Multi-class classification

Companion results for the 5-class case are summarized in the classification report in Table 2. Here we can see that three structure-free encodings are all capable of achieving the more difficult classification task, dealing with greater class imbalance using the three different model architectures. As we saw for the binary classification, greater accuracy, precision and recall is achieved with more sophisticated encoding. Mendeleev+ encoding again delivers perfect results, including the learning curves converging with fewer training instances and the AUC-ROC curve showing perfect TP and FP rates for DTs and SVMs.

**Table 2:** Multi-class classification report for logistics regression (LR), decision trees (DT) and support vector machines (SVM) tested on the computational battery compounds data set, encoded using one-hot, Mendeleev and Mendeleev+ encoding

Algorithm	Encoding	Metric	Class 0	Class 1	Class 2	Class 3	Class 4
		Precision	0.971	0.987	1.000	0.974	0.991
	One-hot	Recall	0.973	0.984	0.992	1.000	0.983
		Accuracy	0.972	0.986	0.996	0.987	0.987
		Precision	0.989	1.000	0.955	0.950	0.975
LR	Mendeleev	Recall	0.985	0.980	0.980	1.000	0.965
		Accuracy	0.987	0.990	0.968	0.974	0.970
		Precision	1.000	1.000	1.000	1.000	1.000
	Mendeleev+	Recall	1.000	1.000	1.000	1.000	1.000
		Accuracy	1.000	1.000	1.000	1.000	1.000
		Precision	0.990	0.995	1.000	0.982	0.982
	One-hot	Recall	0.983	0.995	1.000	1.000	0.991
		Accuracy	0.987	0.995	1.000	0.991	0.987
		Precision	0.998	1.000	0.997	1.000	0.990
DT	Mendeleev	Recall	1.000	0.983	1.000	1.000	0.997
		Accuracy	0.999	1.000	0.990	1.000	0.993
		Precision	1.000	1.000	1.000	1.000	1.000
	Mendeleev+	Recall	1.000	1.000	1.000	1.000	1.000
		Accuracy	1.000	1.000	1.000	1.000	1.000
		Precision	0.993	0.997	1.000	0.991	0.983
	One-hot	Recall	0.993	0.996	1.000	1.000	0.991
		Accuracy	0.993	0.997	1.000	0.996	0.987
		Precision	0.998	0.990	0.994	1.000	0.991
SVM	Mendeleev	Recall	0.998	1.000	0.986	1.000	0.995
		Accuracy	0.998	0.995	0.990	1.000	0.993
		Precision	1.000	1.000	1.000	1.000	1.000
	Mendeleev+	Recall	1.000	1.000	1.000	1.000	1.000
		Accuracy	1.000	1.000	1.000	1.000	1.000

The accuracy is measured using the F1-score

**Fig. 4:**
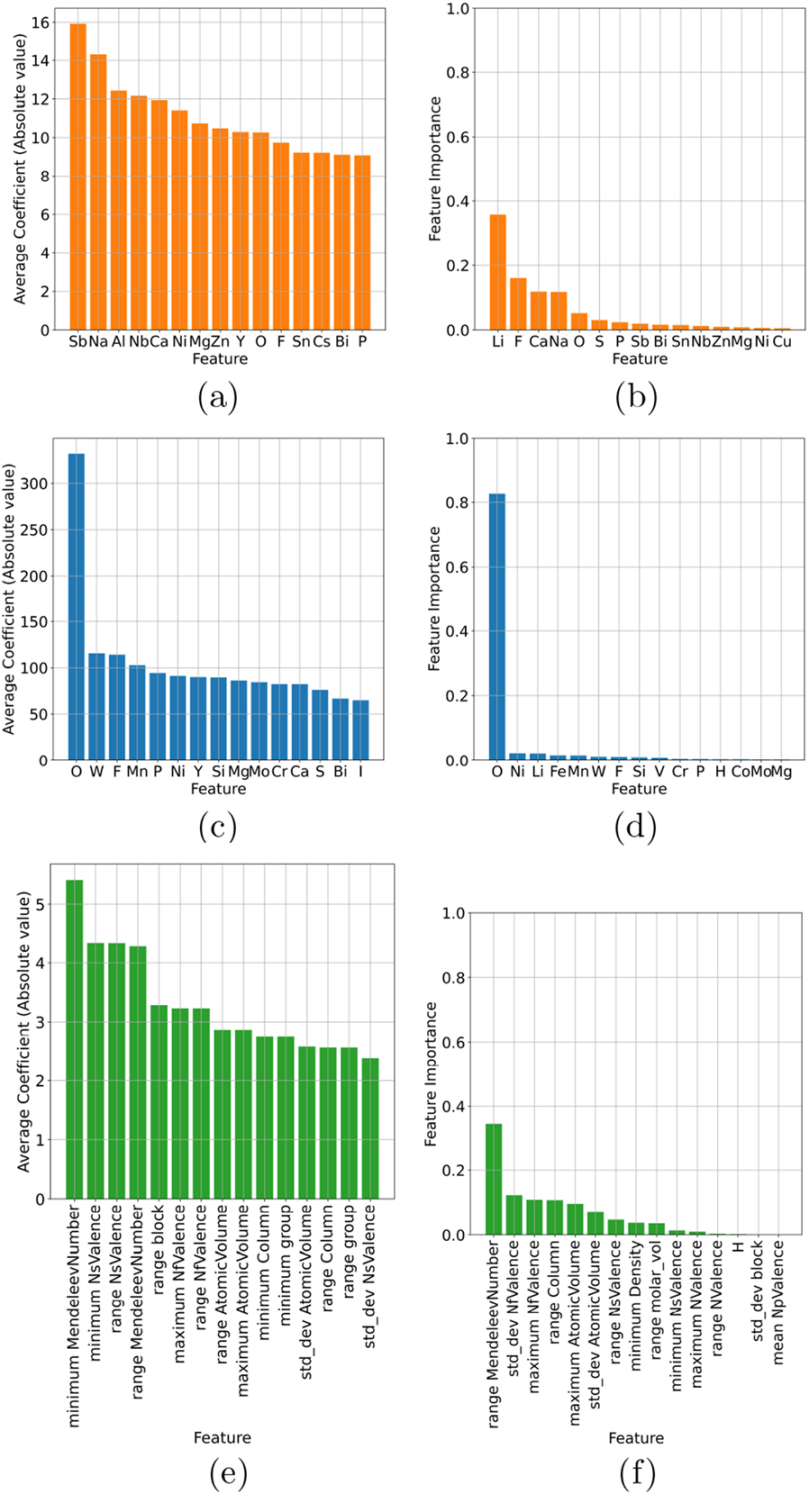
Feature importance profiles showing the top 15 features for multi-class classification of battery compounds in the computational data set using (**a**, **c**, **e**) logistic regression, and (**b**, **d**, **f**) decision trees, encoded using (**a**, **b**) one-hot, (**c**, **d**) Mendeleev, and (**e**, **f**) Mendeleev+ encoding

**Fig. 5:**
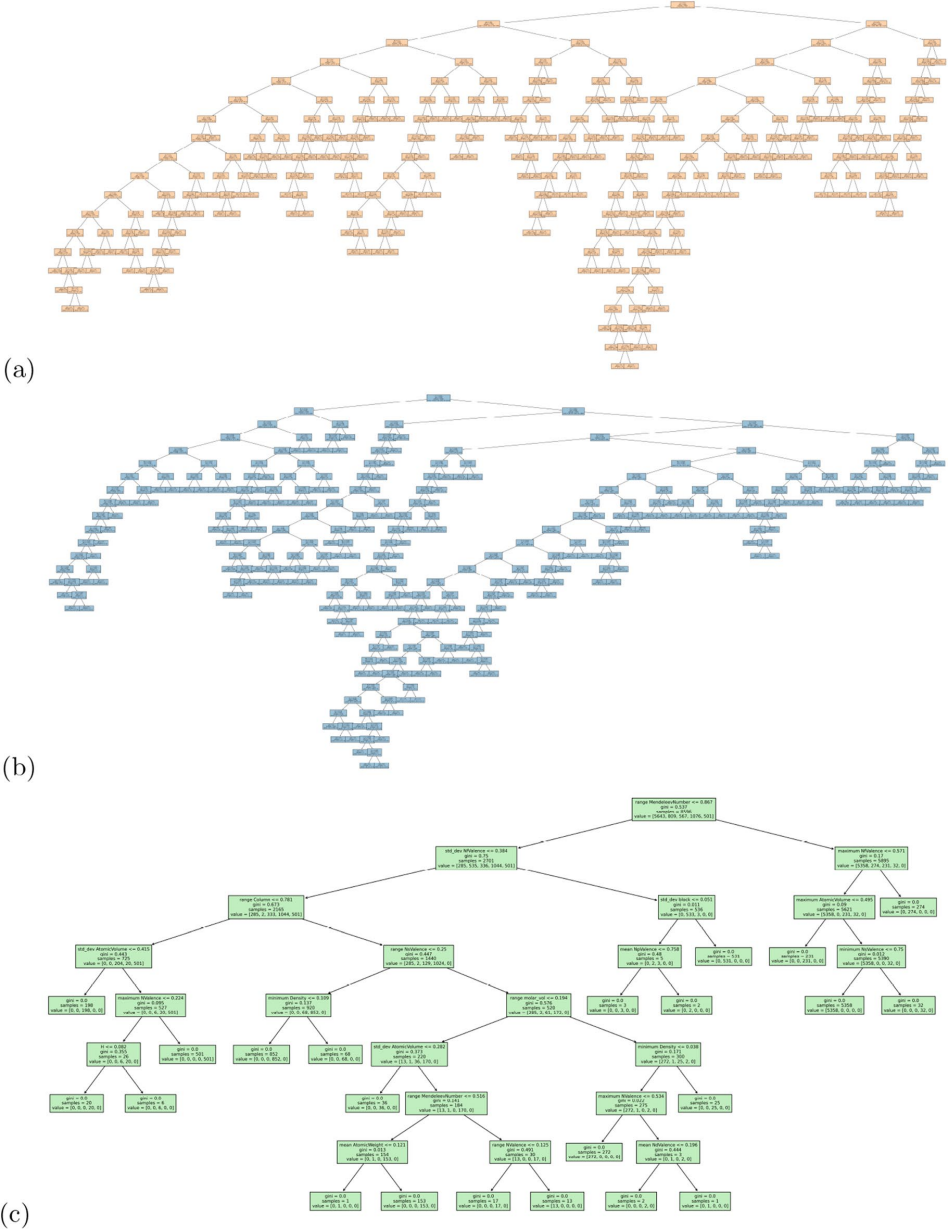
Multi-class classification decision trees for battery compounds in the computational data set, encoded using (**a**) one-hot, (**b**) Mendeleev, and (**c**) Mendeleev+ encoding

When examining the architecture of the interpretable models we also see a similar result to the binary classification, with one-hot encoding producing inconsistent rankings of the elemental features (see Fig. 4a, b) and a deep complicated tree (Fig. 5a); Mendeleev encoding producing relatively consistent rankings of the elemental features (Fig. 4c, d) and a deep, but less complicated tree with multiple leaves on a branch, but fewer branches (Fig. 5b); and Mendeleev+ encoding producing relatively consistent rankings of the summary statistics with fewer insights for battery design (Fig. 4e, f) and a shallow, less complicated tree (Fig. 5c). Due to the improved efficiency over one-hot encoding, and the superior insights over Mendeleev+ encoding, and the high precision, recall, accuracy and sensitivity (see AUC-ROC curves in Additional file 1), Mendeleev encoding is recommended for structure-free classification.

### Experimental observations

The exploration above compared the three encodings with two tasks using three different classifiers based on three different logics, evaluated with the 4 different metrics concluded that Mendeleev encoding is superior. This is a comprehensive comparison, also identified variations in model complexity and efficiency, and the inferiority of Mendeleev+ interpretability.

To challenge the utility of Mendeleev encoding in classification tasks, we applied the same set of test algorithms and metrics to a much larger and more challenging experimental data set. This data set consists of 265481 data instances of battery materials, refined from the work of Huang and Cole [18]. Each data instance represents a battery cell that contains one or more battery components, with each component expressed as its chemical formula. The set was originally 311716 chemical formulas auto-extracted from the literature, but due to inaccurate document digitisation and error-prone chemical language processing significant data cleaning and processing was required. We developed a set of sophisticated rules to inspect the formulas, and data instances are either retained, corrected, or discarded accordingly, to minimize errors.

An electronic notebook outlining this procedure is included in Additional files 2 (using Additional file 3), but in short, instances are dropped when there are non-numeric coefficients in the formulas (e.g. SiO_x_); one of the components listed by the original paper is an ion (e.g. Ni(II) or Ni2^+^); and when one of the formulas is manually identified as invalid due to text recognition errors. Common mistakes include Oxygen being confused with zero, capital I and/or lowercase l being confused with number one, missing battery components due to the ChemDataExtractor not recognising “–” (dash) as a separator, or one of the formulas being manually identified as erroneous when compared to the source text.

**Fig. 6:**
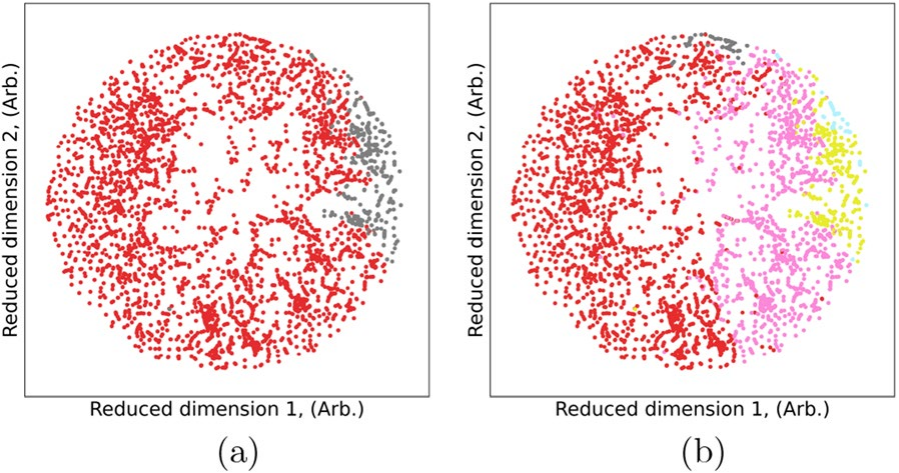
Visualisation the results of agglomerative clustering of battery compounds in the experimental data set, encoded using Mendeleev encoding, showing (**a**) 2 clusters, and (**b**) 5 clusters

This data set was also processed using agglomerate clustering and the binary and multi-class (5 class) cases are visualized in Fig. 6. We applied LR, DT and SVM classifiers to these categorical labels, and the classification report is provided in Table 3. The model parameters are listed in Additional file 1. Once again, Mendeleev encoding has represented the materials, which are linearly separable, with outstanding precision, recall and accuracy (see confusion matrices with TP, TN, FP, FN in Additional file 1), with excellent sensitivity (TP and FP rates) in the AUC-ROC curves in Additional file 1.

**Table 3:** Multi-class classification report for logistics regression (LR), decision trees (DT) and support vector machines (SVM) tested on the experimental battery compounds data set, encoded using Mendeleev encoding

Classification	Algorithm	Metric	Class 0	Class 1	Class 2	Class 3	Class 4
		Precision	1.000	1.000	—	—	—
	LR	Recall	1.000	0.997	—	—	—
		Accuracy	1.00	0.998	—	—	—
		Precision	1.000	1.000	—	—	—
Binary	DT	Recall	1.000	1.000	—	—	—
		Accuracy	1.000	1.000	—	—	—
		Precision	1.000	1.000	—	—	—
	SVM	Recall	1.000	0.998	—	—	—
		Accuracy	1.000	0.999	—	—	—
		Precision	0.996	0.996	0.992	1.000	0.999
	LR	Recall	0.998	0.992	0.992	1.000	0.996
		Accuracy	0.997	0.994	0.992	1.000	0.997
		Precision	1.000	0.999	0.996	0.995	1.000
Multi-class	DT	Recall	1.000	1.000	1.000	1.000	0.999
		Accuracy	1.000	1.000	0.998	0.997	1.000
		Precision	1.000	1.000	1.000	1.000	1.000
	SVM	Recall	1.000	1.000	0.988	0.989	0.995
		Accuracy	1.000	1.000	0.994	0.999	0.997

The accuracy is measured using the F1-score

**Fig. 7:**
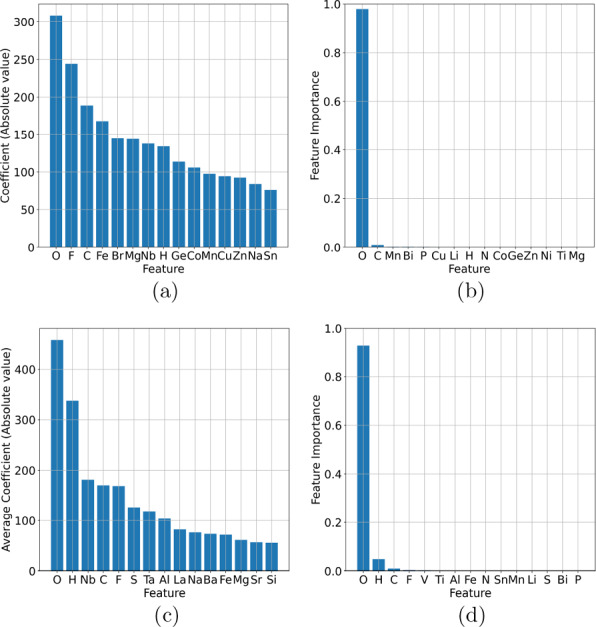
Feature importance profiles showing the top 15 features of battery compounds in the experimental data set, encoded using Mendeleev encoding, for binary classification using **a** logistic regression, and **b** decision tree classification; and multi-class classification using **c** logistic regression, and **d** decision tree classification

**Fig. 8:**
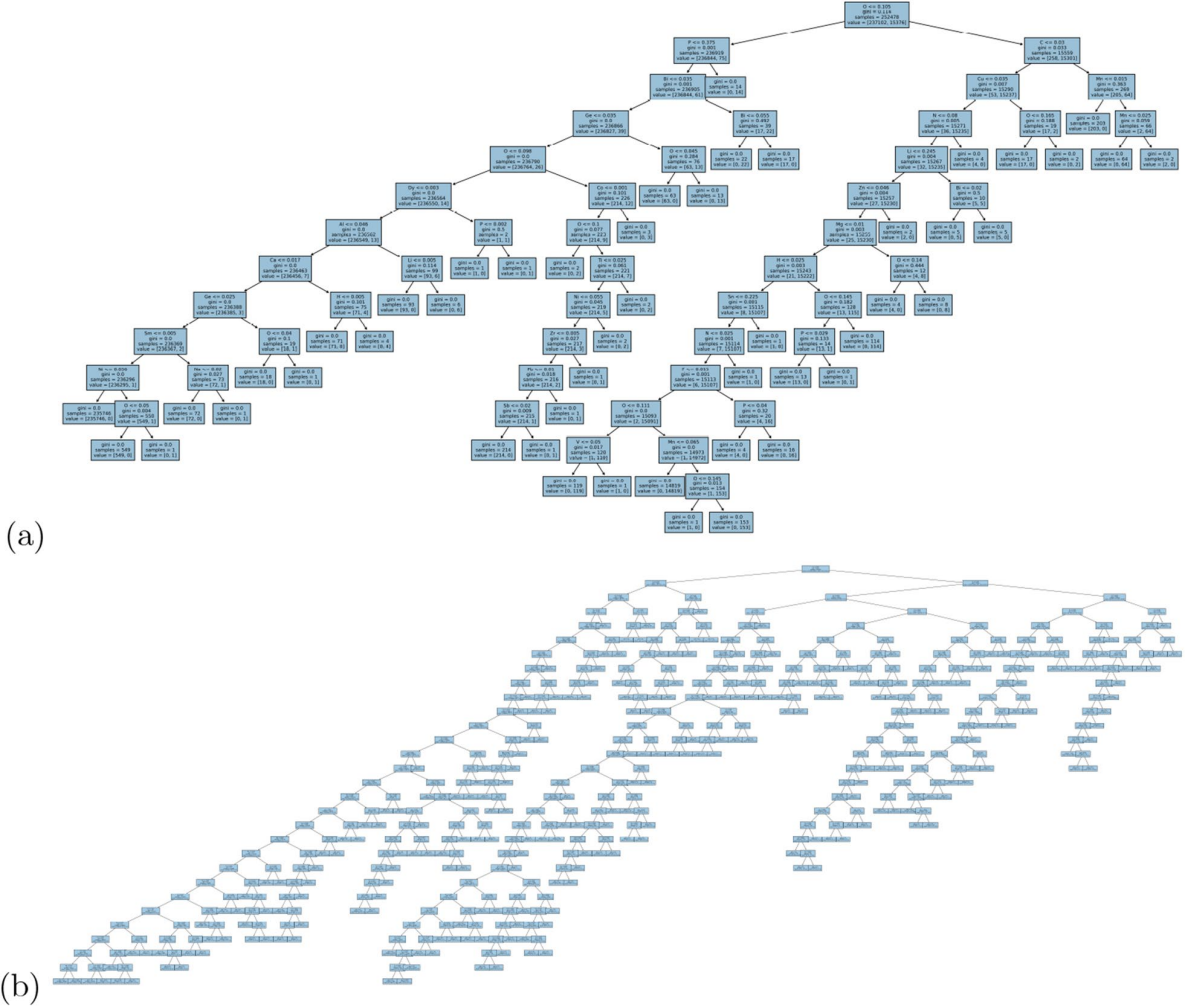
**a** Binary decision tree, and **b** multi-class decision tree for battery compounds in the experimental data set, encoded using Mendeleev encoding

We can also see that the feature importance histograms are remarkably consistent (see Fig. 7. Both LR and DT report O as the most important element in the model, with H, F, and C also consistently among the top 4 (occupying the upper nodes in the decision trees in Fig 8), for both the binary and multi-class schemes. This consistency gives confidence that the concentration of these elements are critical to determining the class of battery compounds.

## Discussion

The results above confirm the reliability of Mendeleev encoding for binary and multi-class separation tasks using three algorithms, based of convectional evaluation metrics such as precision, recall, accuracy (see Tables 1,  2 and 3) and ROC-AUC sensitivity (see Additional file 1). The scores are consistently over 95% for LR, 98% for DT and 99% for SVM, even with imbalanced classes. This detailed comparison also revealed additional advantages in terms of efficiency and interpretability by using the more expressive and informative features of Mendeleev encoding.

**Fig. 9:**
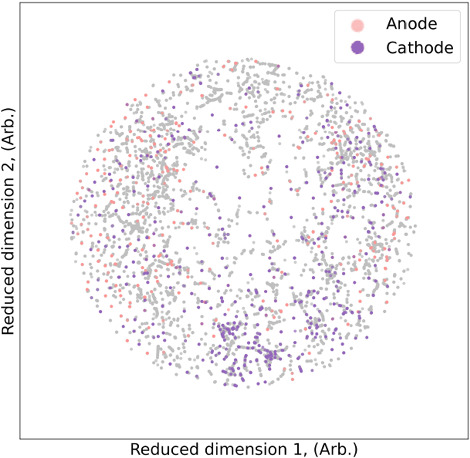
Distribution of the anode and cathode materials across the experimental data set, bearing no relations to the classes confirmed from unsupervised learning show in Fig. 6

Having established that Mendeleev encoding provides an effective way to separate complex battery compounds without requiring structural information, the question remains as to how well these classes reflect the materials chemistry? To investigate the relationship between the chemical composition of the experimental battery classes and the functional properties, we have again used the manifold t-SNE mapping for visualisation. The results for showing the distribution of anode and cathode materials are show in Fig. 9, which can be directly compared with Fig. 6. As we can see from this comparison, the applications as anodes or cathodes has no relation to either the binary or multiple classes. These classes are intrinsic, though there is evidence for some minor grouping of cathode and anode materials in Fig. 9.

**Fig. 10:**
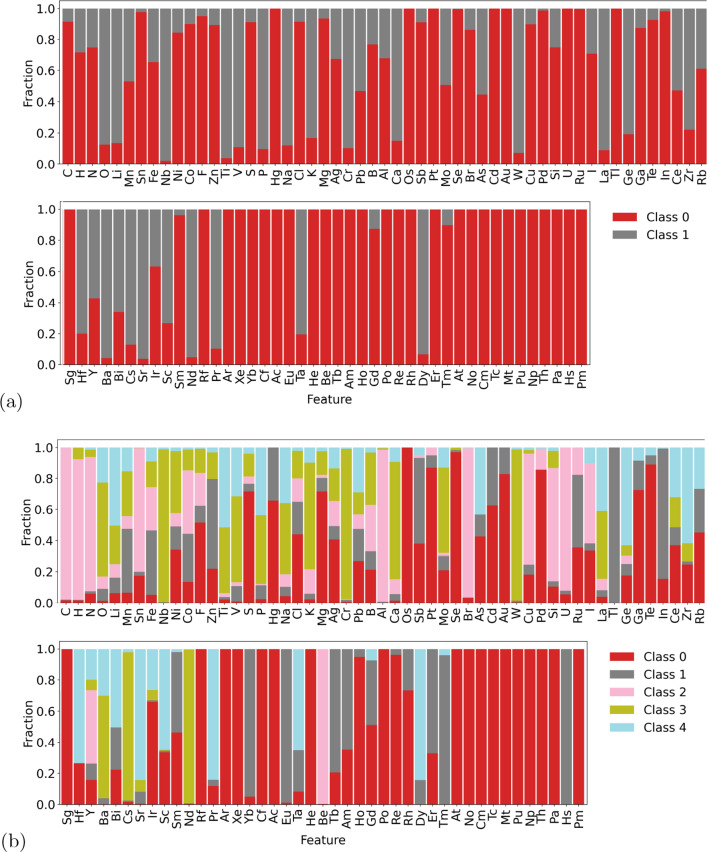
Normalized average features values for each class in the experimental battery compounds, separated using **a** binary, and **b** multi-class classification with Mendeleev encoding

The composition of the classes can be distinguished by comparing the normalized average features values for each class, as shown in Fig. 10. In the case of the binary classification in Fig. 10a we can see that there are a large number of elements that only appear in Class 0, including many Lanthanides and Actinides. Class 0 also has materials with higher concentrations of C, H, and N, while Class 1 has much higher concentrations of O, Li, Ti, V and P. The distributions between classes change when 5 classes are separated (Fig. 10b), but similarities with the binary classification can be observed, such as Class 2 containing the highest concentrations of C, H and N, and Class 0 containing most Lanthanides and Actinides.

While there are multiple ways of encoding materials compounds for machine learning applications, Mendeleev encoding is straightforward, scientifically intuitive, computationally efficient and accurate. Although Mendeleev+ produces more accurate models than Mendeleev encoding when using simpler algorithms like LR, the performance difference quickly diminishes when the complexity of the classifiers rises: both were able to achieve perfect results using SVM. This could be explained by the fact that all additional features in Mendeleev+ can be derived from Mendeleev encoding, and Mendeleev+ can be seen as a transformed feature space by using a fixed mapping function on Mendeleev encoding, which is not unlike the kernel transformation in SVM.

Mendeleev encoding can be performed using widely available informatics platforms, but this study represents the first rigorous test that confirms the validity of the approach for classification tasks, from both a domain and data perspective.

## Conclusions

Determining the precise structure of a materials is expensive; either in terms of (human or computer) time, resources, expertise or infrastructure. The ability to explore a materials space before investment of structural characterisation has been made has scientific and economic advantages.

In this study we have evaluated the use of three structure-free materials encodings, with increasing chemical complexity, for two different supervised classification tasks (binary and multi-class classification). Using a computational data set of battery compounds we have compared one-hot, Mendeleev and Mendeleev+ encoding using three different linear and non-linear classifiers based on different logics, and evaluated their performance using learning curves, precision, recall, accuracy and area under the receiver operating characteristic curves to test their sensitivity. We also used the feature importance rankings exposed by the interpretable classifiers to show how the different encoding affect the model architectures. The decision trees produced by the DT algorithms were shown, and the materials data were visualised using manifold learning and feature value histograms.

Through this comprehensive comparison we find that the Mendeleev encoding provides the best balance between model complexity and performance. This encoding, which decomposes the chemical formula into features representing the concentration of elements in the periodic table, is able to accurately support binary and multi-class classification using logistic regression, decision trees and support vector machines, for highly complex materials compounds with superior consistency and interpretability. The scores are consistently over 95% for LR, 98% for DT and 99% for SVM, even with imbalanced classes.

Future work is planned to determine if this performance extends to the prediction of continuous material properties using regression, and to other complex materials such as alloys, where the macroscopic performance is known to be intrinsically link to the composition, even at low elemental concentrations.

### Supplementary Information

**Additional file 1.** This document contains additional classification results on data sets of battery compounds encoded using one-hot, Mendeleev and Mendeleev+ encoding. This includes learning curves, confusion matrices and AUC-ROC curves using logistic regression, decision trees and support vector machines, for binary classification (Figs. S1, S2 and S3 for the one-hot, Mendeleev and Mendeleev+ encoded computational data, respectively), multiple classification (Figs. S4, S5 and S6 for the one-hot, Mendeleev and Mendeleev+ encoded computational data, respectively). In addition to this the learning curves, confusion matrices and AUC-ROC curves using logistic regression, decision trees and support vector machines, for binary classification (Fig. S7) and multiple classification (Fig. S8) of the Mendeleev encoded experimental data set. All model parameters for both data sets are provided in Tables S4, S2, S3, S5 and S6.

**Additional file 2.** Electronic notebook to undertake cleaning of the experimental data set, in combination with Additional file 3.

**Additional file 3.** Periodic table csv file to undertake cleaning of the experimental data set, in combination with Additional file 2.

## Data Availability

No datasets were generated or analysed during the current study.
