# Supplementary material for: Classification of battery compounds using structure-free Mendeleev encodings
Source: J Cheminform. 2024 Apr 26;16:47. doi: 10.1186/s13321-024-00836-x (PMC11055346; doi:10.1186/s13321-024-00836-x)
Supplement: Supplementary file 1 — Additional file 1. This document contains additional classification results on data sets of battery compounds encoded using one-hot, Mendeleev and Mendeleev+ encoding. This includes learning curves, confusion matrices and AUC-ROC curves using logistic regression, decision trees and support vector machines, for binary classification (Figs. S1, S2 and S3 for the one-hot, Mendeleev and Mendeleev+ encoded computational data, respectively), multiple classification (Figs. S4, S5 and S6 for the one-hot, Mendeleev and Mendeleev+ encoded computational data, respectively). In addition to this the learning curves, confusion matrices and AUC-ROC curves using logistic regression, decision trees and support vector machines, for binary classification (Fig. S7) and multiple classification (Fig. S8) of the Mendeleev encoded experimental data set. All model parameters for both data sets are provided in Tables S4, S2, S3, S5 and S6. [file 13321_2024_836_MOESM1_ESM.pdf]

SUPPORTING INFORMATION
Classification of Battery Compounds using Structure-free Mendeleev Encodings

Zixin Zhuang[1] and Amanda S. Barnard*[1]

[1]*School of Computing, Australian National University, Acton 2601, Australia*

This document contains additional classification results on data sets of battery compounds encoded using one-hot, Mendeleev and Mendeleev+ encoding. This includes learning curves, confusion matrices and AUC-ROC curves using logistic regression, decision trees and support vector machines, for binary classification (Figs. S1, S2 and S3 for the one-hot, Mendeleev and Mendeleev+ encoded computational data, respectively), multiple classification (Figs. S4, S5 and S6 for the one-hot, Mendeleev and Mendeleev+ encoded computational data, respectively). In addition to this the learning curves, confusion matrices and AUC-ROC curves using logistic regression, decision trees and support vector machines, for binary classification (Fig. S7) and multiple classification (Fig. S8) of the Mendeleev encoded experimental data set. All model parameters for both data sets are provided in Tables S4, S2, S3, S5 and S6.

*amanda.s.barnard@anu.edu.au

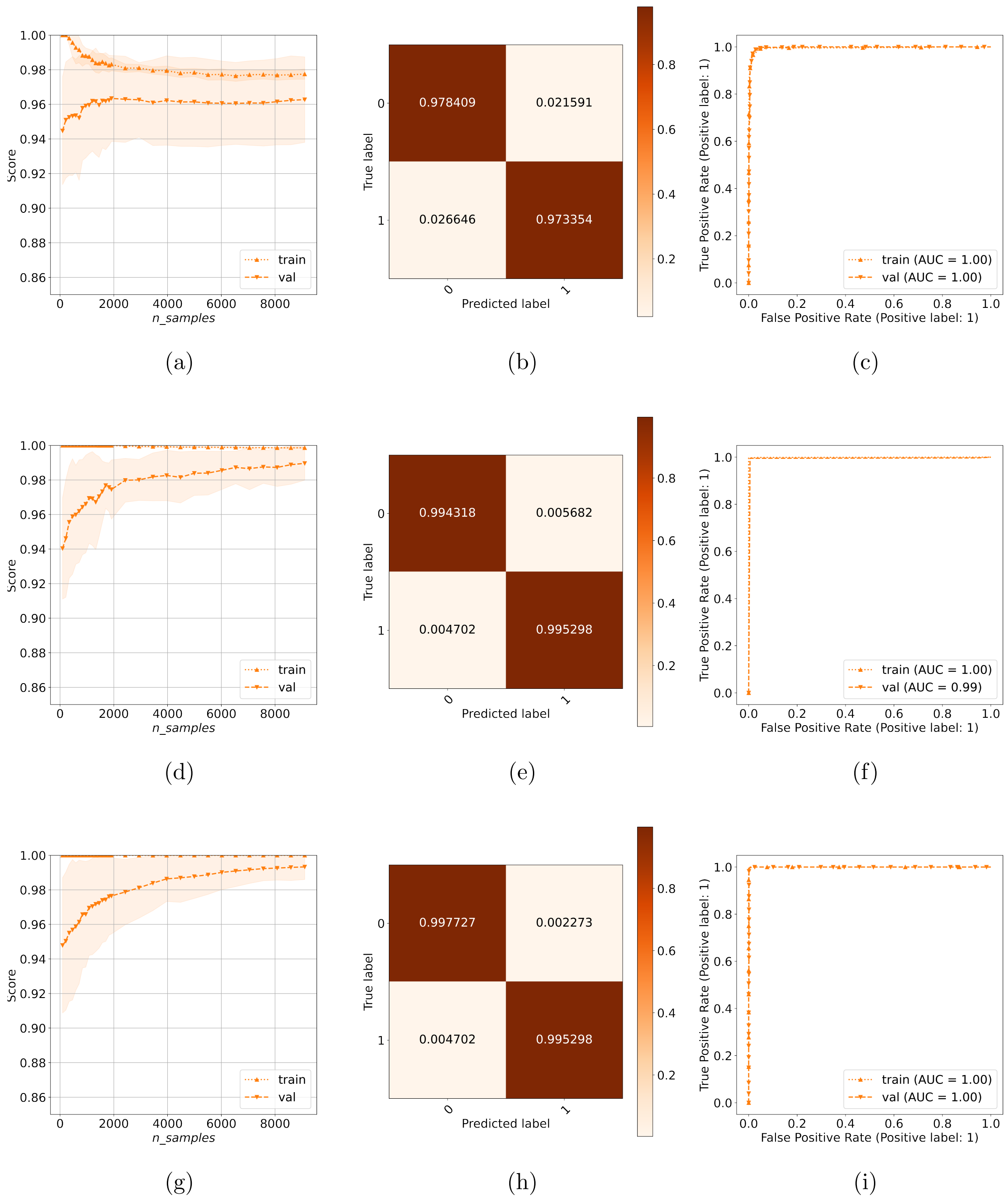

Figure S1: Binary classification of battery compounds in the computational data set, encoded using one-hot encoding, and comparing the learning curves for the training set and validation sets (a, d, g), the confusion matrix for the testing set (b, e, h) and the area under the receiver operating characteristic curve (AUC-ROC) for the training and validation sets, generated using logistic regression (top), decision tree classification (middle) and support vector classification (bottom).

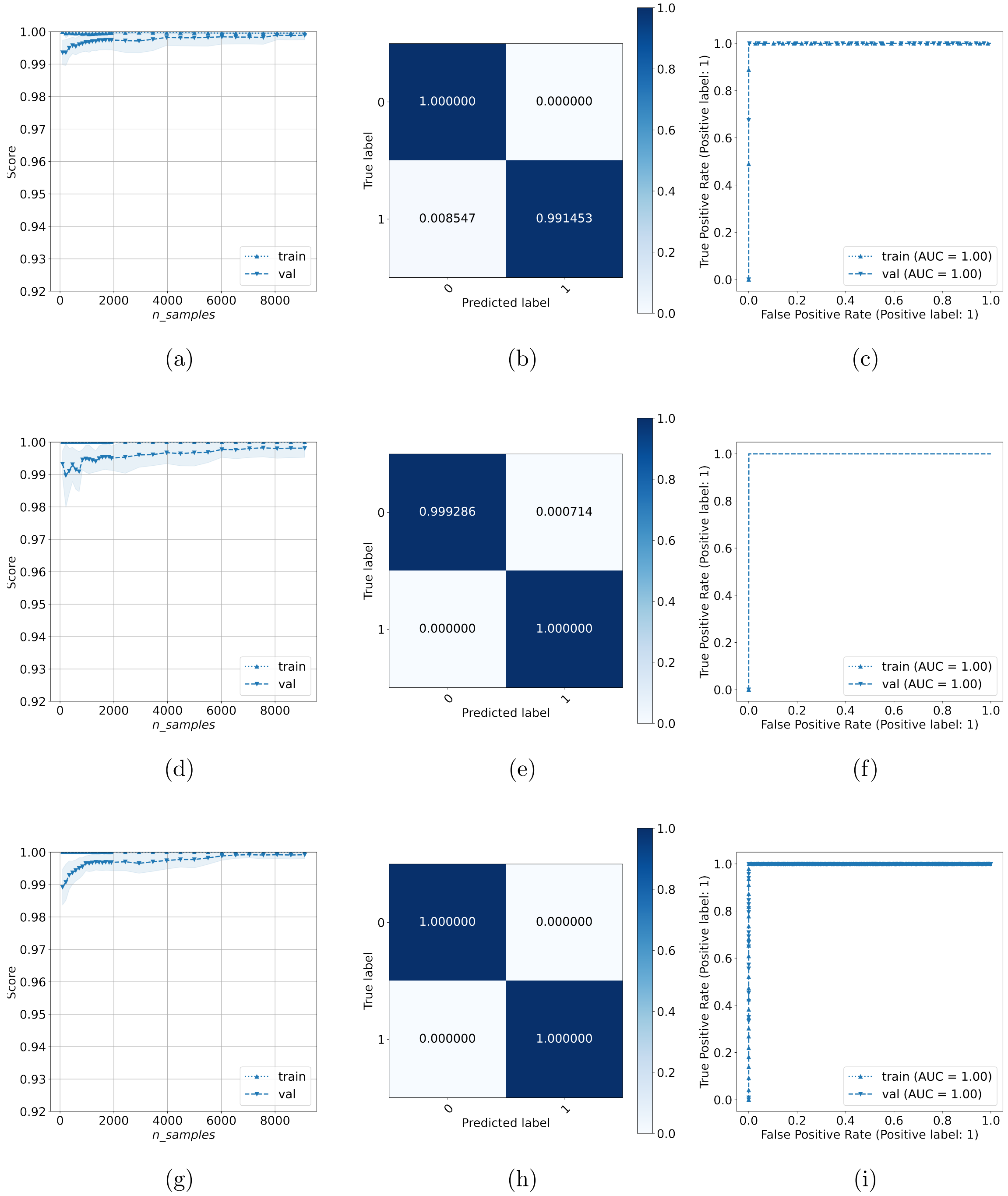

Figure S2: Binary classification of battery compounds in the computational data set, encoded using one-hot encoding, and comparing the learning curves for the training set and validation sets (a, d, g), the confusion matrix for the testing set (b, e, h) and the area under the receiver operating characteristic curve (AUC-ROC) for the training and validation sets, generated using logistic regression (top), decision tree classification (middle) and support vector classification (bottom).

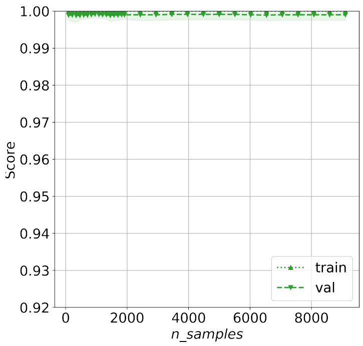

(a)

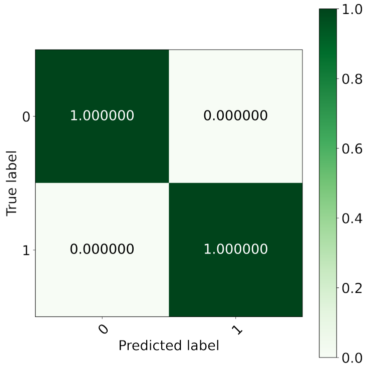

(b)

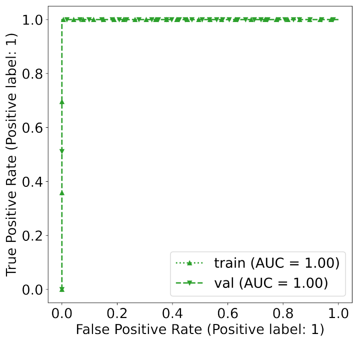

(c)

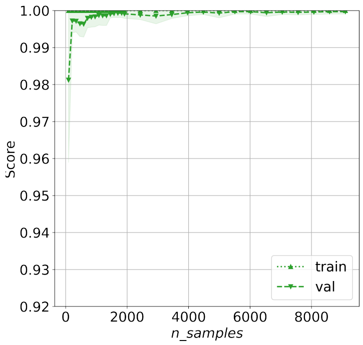

(d)

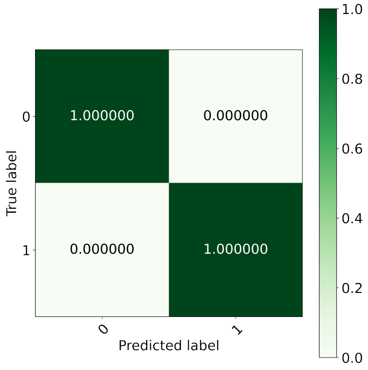

(e)

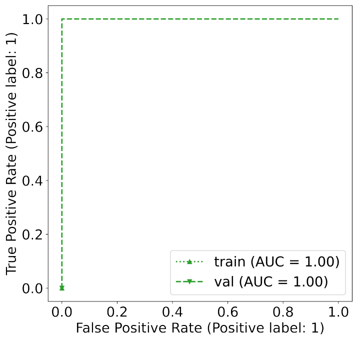

(f)

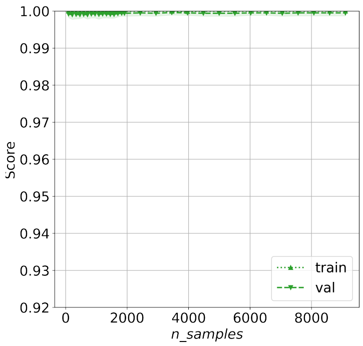

(g)

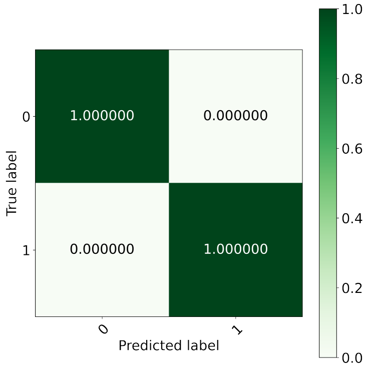

(h)

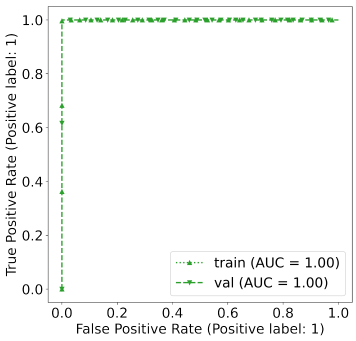

(i)

Figure S3: Binary classification of battery compounds in the computational data set, encoded using one-hot encoding, and comparing the learning curves for the training set and validation sets (a, d, g), the confusion matrix for the testing set (b, e, h) and the area under the receiver operating characteristic curve (AUC-ROC) for the training and validation sets, generated using logistic regression (top), decision tree classification (middle) and support vector classification (bottom).

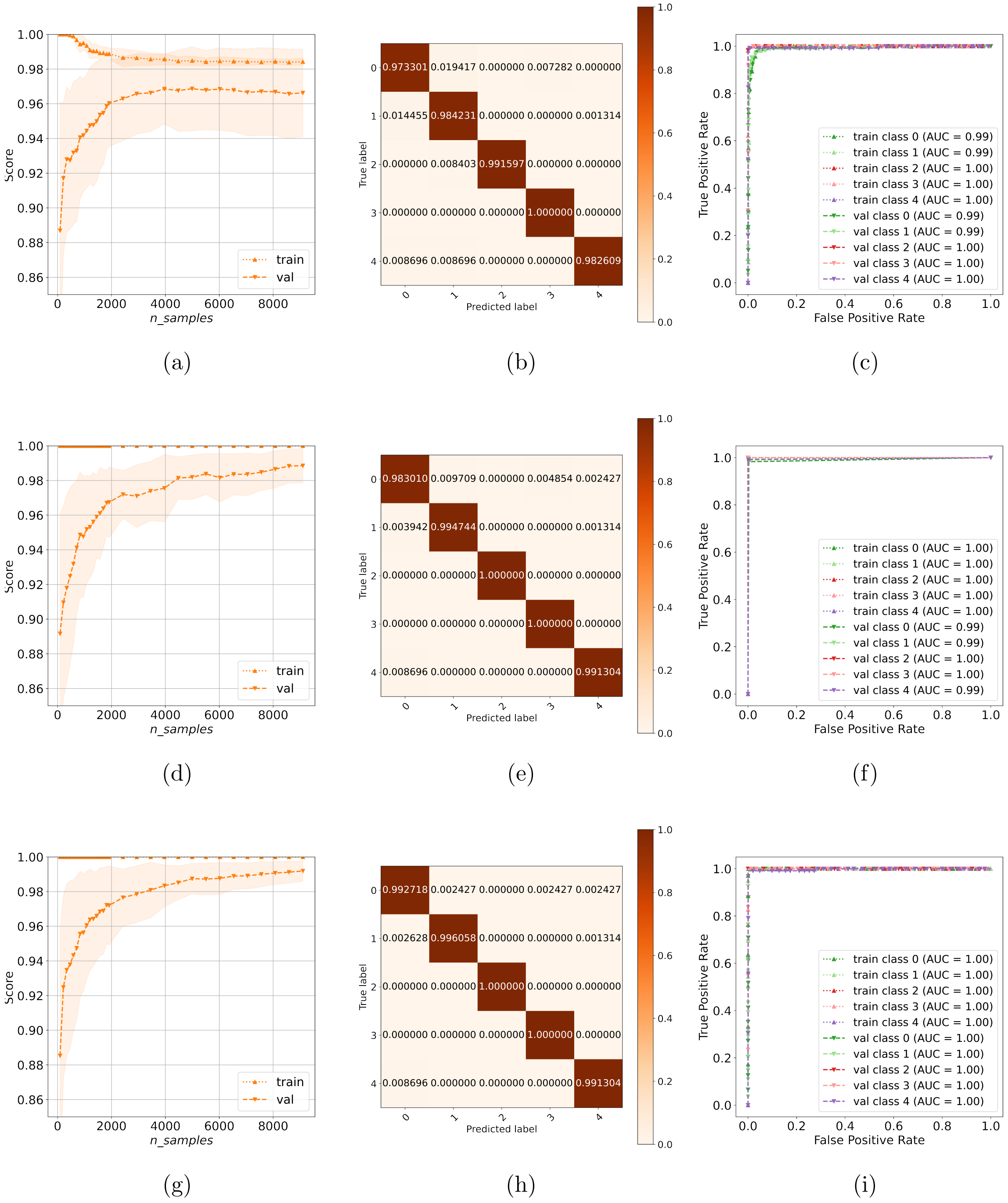

Figure S4: Multi-class classification of battery compounds in the computational data set, encoded using one-hot encoding, and comparing the learning curves for the training set and validation sets (a, d, g), the confusion matrix for the testing set (b, e, h) and the area under the receiver operating characteristic curve (AUC-ROC) for the training and validation sets, generated using logistic regression (top), decision tree classification (middle) and support vector classification (bottom).

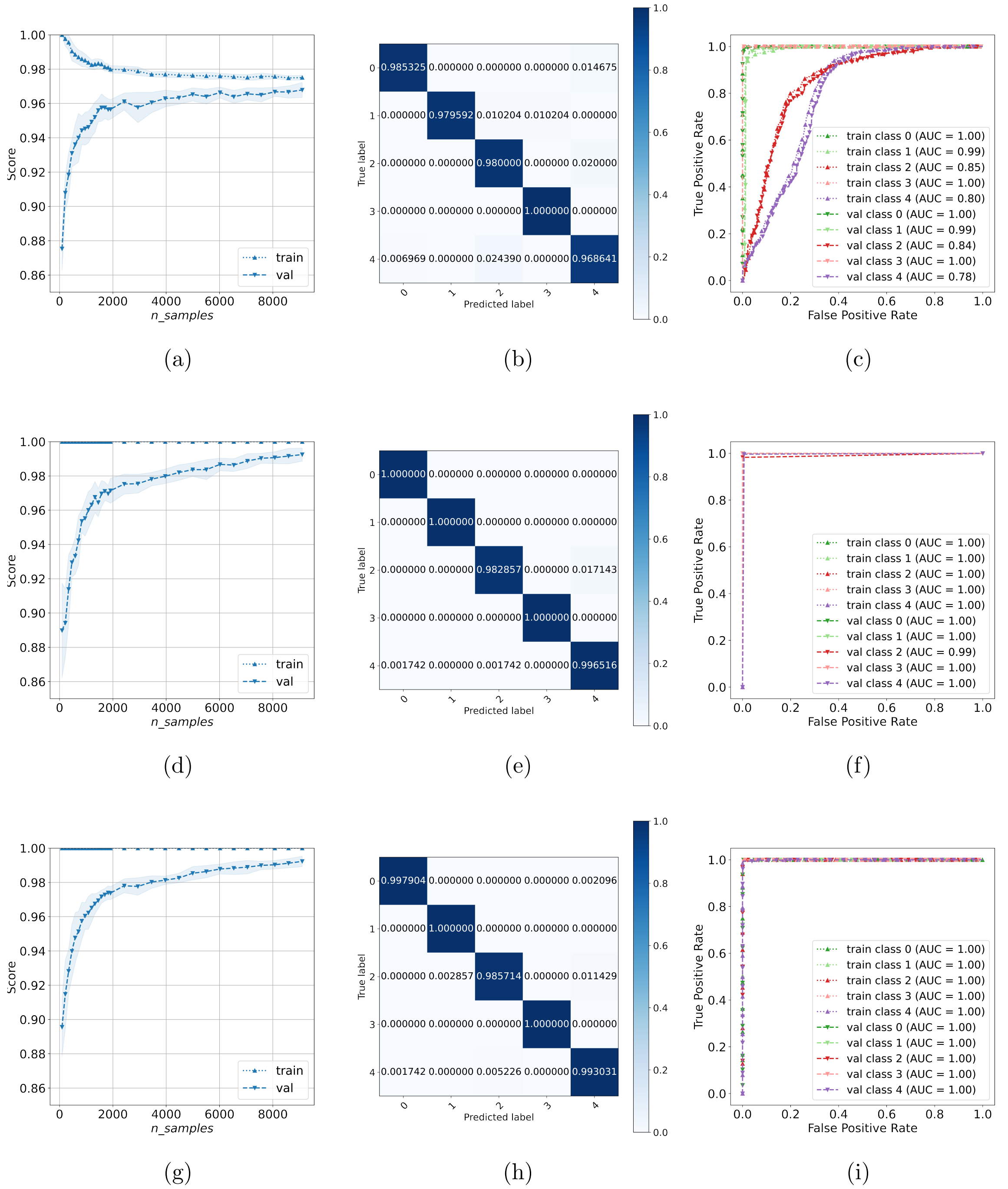

Figure S5: Multi-class classification of battery compounds in the computational data set, encoded using Mendeleev encoding, and comparing the learning curves for the training set and validation sets (a, d, g), the confusion matrix for the testing set (b, e, h) and the area under the receiver operating characteristic curve (AUC-ROC) for the training and validation sets, generated using logistic regression (top), decision tree classification (middle) and support vector classification (bottom).

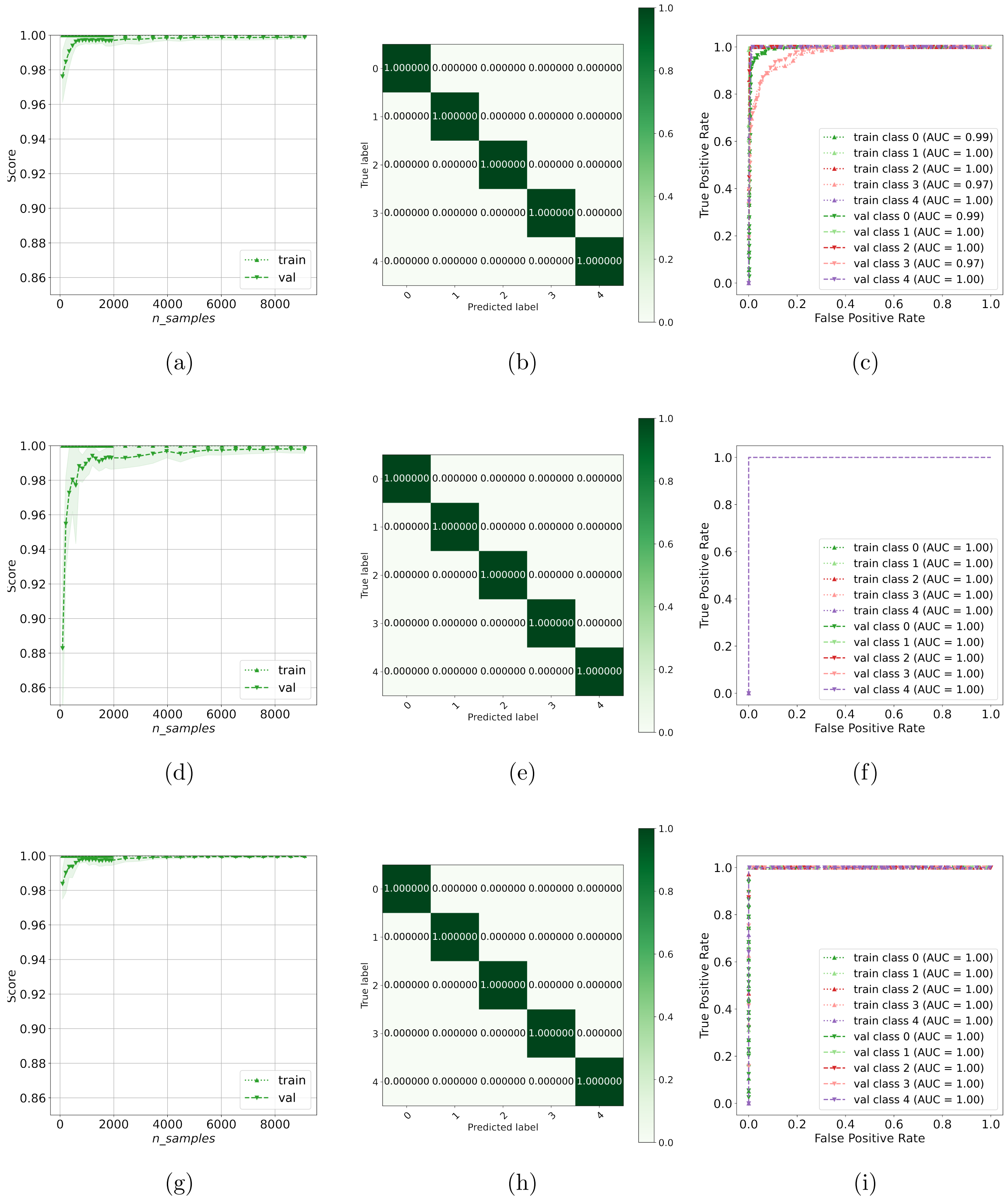

Figure S6: Multi-class classification of battery compounds in the computational data set, encoded using Mendeleev+ encoding, and comparing the learning curves for the training set and validation sets (a, d, g), the confusion matrix for the testing set (b, e, h) and the area under the receiver operating characteristic curve (AUC-ROC) for the training and validation sets, generated using logistic regression (top), decision tree classification (middle) and support vector classification (bottom).

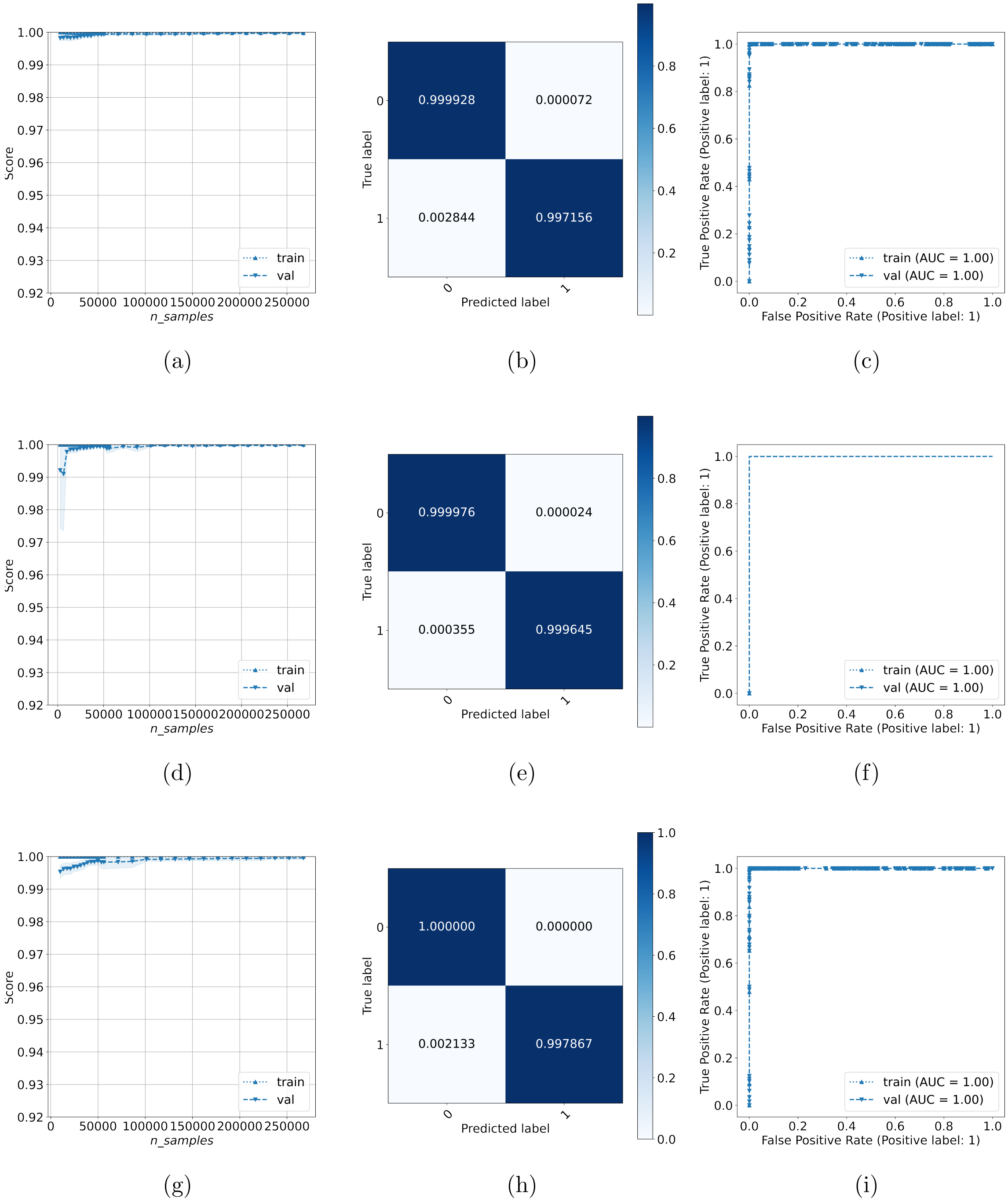

Figure S7: Binary classification of battery compounds in the experimental data set, encoded using Mendeleev encoding, and comparing the learning curves for the training set and validation sets (a, d, g), the confusion matrix for the testing set (b, e, h) and the area under the receiver operating characteristic curve (AUC-ROC) for the training and validation sets, generated using logistic regression (top), decision tree classification (middle) and support vector classification (bottom).

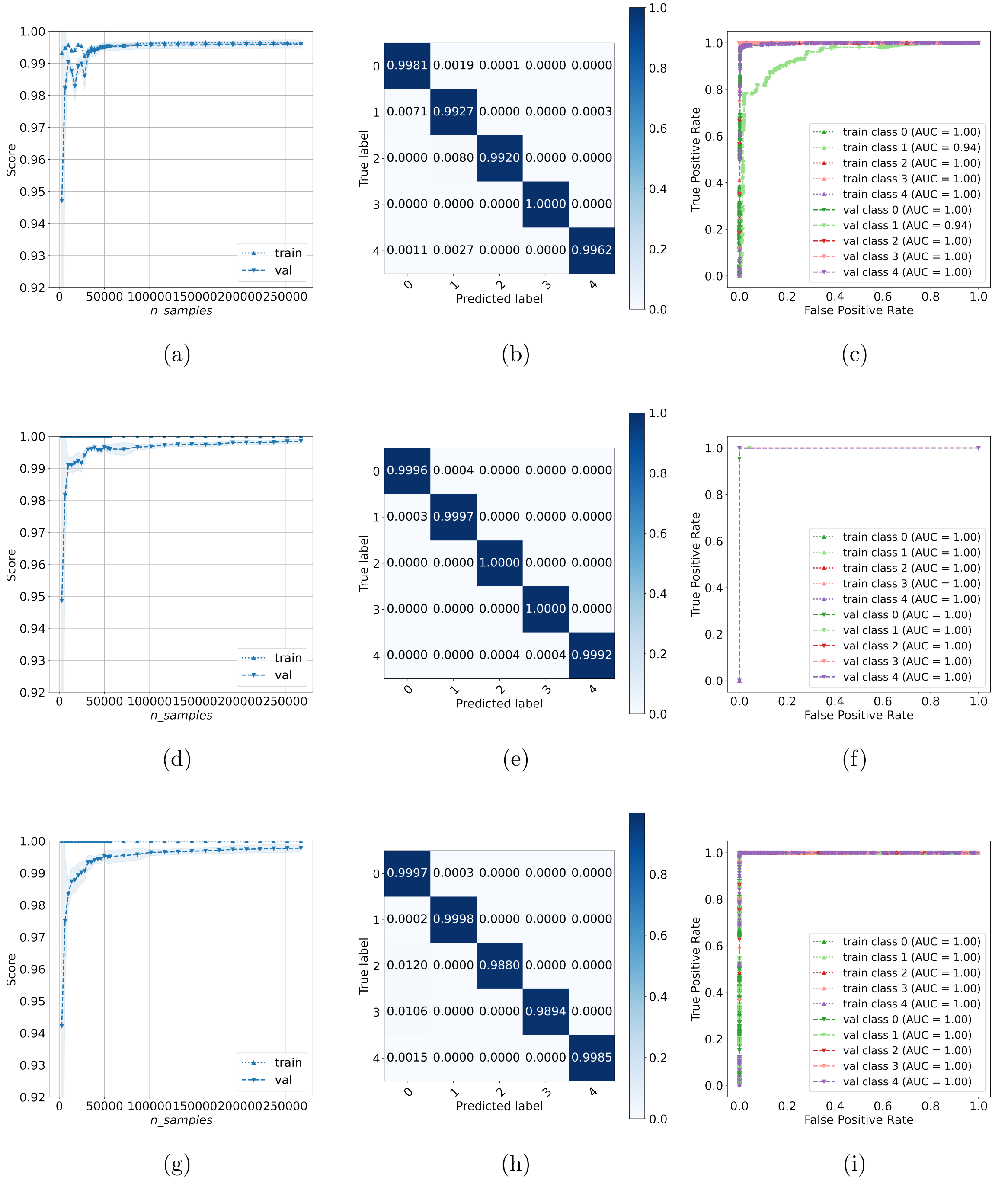

Figure S8: Multi-class classification of battery compounds in the experimental data set, encoded using Mendeleev encoding, and comparing the learning curves for the training set and validation sets (a, d, g), the confusion matrix for the testing set (b, e, h) and the area under the receiver operating characteristic curve (AUC-ROC) for the training and validation sets, generated using logistic regression (top), decision tree classification (middle) and support vector classification (bottom).

Table S1: The optimized hyperparameters of LR models for the computational data set. C is the regularization parameter, and is inversely proportional to the strength of L2 regularization.

Encoding	*n_classes*	C
One-hot	2	200
	5	1439
Mendeleev	2	1500
	5	54556
Mendeleev+	2	207
	5	50

Table S2: The optimized hyperparameter of DT models for the computational data set. *max_depth* indicates the maximum depth of the resultant decision tree. If *max_depth* is set to None (no tree depth regularisation), the actual depth of the constructed tree is reported.

Encoding	*n_classes*	*max_depth* (actual depth)
One-hot	2	15
	5	None (19)
Mendeleev	2	10
	5	None (28)
Mendeleev+	2	None (3)
	5	None (8)

Table S3: The optimized hyperparameter of SVM models for the computational data set. C is the regularization parameter, and is inversely proportional to the strength of L2 regularization. Kernel indicates the kernel type used in SVM.

Encoding	*n_classes*	C	Kernel
One-hot	2	10	RBF
	5	100	RBF
Mendeleev	2	100	RBF
	5	2905	RBF
Mendeleev+	2	4.28	RBF
	5	100	RBF

Table S4: The optimized hyperparameters of LR models using Mendeleev encoding for the experimental data set. C is the regularization parameter, and is inversely proportional to the strength of L2 regularization.

n_classes	C
2	3792
5	6951927

Table S5: The optimized hyperparameter of DT models using Mendeleev encoding for the experimental data set. *max_depth* indicates the maximum depth of the resultant decision tree. If *max_depth* is set to None (no tree depth regularisation), the actual depth of the constructed tree is reported.

n_classes	*max_depth* (actual depth)
2	None (14)
5	29

Table S6: The optimized hyperparameter of SVM models using Mendeleev encoding for the experimental data set. C is the regularization parameter, and is inversely proportional to the strength of L2 regularization. Kernel indicates the kernel type used in SVM.

n_classes	C	Kernel
2	100	RBF
5	5000	RBF

batteries-workflow

November 28, 2023

1 General Information

1.1 1. The Dataset

This notebook contains our effort to reformat and clean the experimental dataset of battery materials that is auto-generated using ChemDataExtractor.

Files: - battery_clean.csv: the cleaned dataset - battery.csv: the original dataset by the cambridge group - periodic_table.csv: a periodic table - battery_corrected.csv: the file that contains manual corrections (see Step 3 of the code section)

The clean version of the dataset has 266467 out of 292313 datapoints of the original dataset, each representing a battery cell. Each datapoint records exactly one property of the battery cell. There are 26 features/columns in the dataset:

Feature	Description
Formulas	The formulas of battery components as a list of strings
Name_extracted	The components extracted by ChemdataExtractor as a list of dictionaries
Name_source	Text extracted from the original paper that contains information about battery composition
heterojunction	Whether or not the battery has a dash separator that may imply heterojuntion in its Name_source
heterojunction_unresolved	If the Formulas has not been corrected with regard to heterojunction (see info about errors)
DOI	DOI number of the original paper
Date	Date of publication. Might be helpful for data cleaning
Warning	Warnings about the datapoint: Serial, near-Limit and Related. See the paper for details
Type of the material	Anode or Cathode
Property	Propery the datapoint describes: voltage, capacity, efficiency, conductivity and Energy
Specifier	Further information about the property
Capacity_is_discharge	Whether or not the recorded capacity is discharge capacity
Capacity_is_charge	Whether or not the recorded capacity is charge capacity
Capacity_is_reversible	Whether or not the recorded capacity is reversible capacity
Capacity_is_specific	Whether or not the recorded capacity is specific capacity
Capacity_is_theoretical	Whether or not the recorded capacity is theoretical capacity
Capacity/Gram$^{(-1.0)Hour}$(1.0)MilliAmpere^(1.0)	Capacity

Feature	Description
Cycles	Cycles
Current/C__rate	Current in C__rate, cleaned to get rid of textual data
Current__original//C__rate	Current in C__rate, contains textual data
Current/mAg^(-1)	Current in mAg^(-1), cleaned to get rid of textual data
Current__original/mAg^(-1)	Current in mAg^(-1), contains textual data
Voltage/Volt^(1.0)	Voltage
Coulombic Efficiency/Percent^(1.0)	Coulombic Efficiency
Conductivity/CentiMeter^(-1.0)___Siemens^(1.0)	Conductivity
Energy/KiloGram^(-1.0)___WattHour^(1.0)	Energy

Please refer to the original paper for further information.

1.2 2. Data Cleaning and Error Correction

The original dataset augmented energy related data by derving new datapoints from existing datapoints using the formula:

Energy (Wh / g) = Capacity (mAh / g) ∗ Voltage (V)/1000

We removed the calculated datapoints as the calculation result might be problematic and inaccurate.

Based on the correctness of the formula, we have removed/ corrected some problematic datapoints.

- Datapoints are dropped when:
  - There are non-numeric coefficients in the formulas (e.g. SiOx)
  - One of the components listed by the original paper is an ion (e.g. Ni(II) or Ni2+). This is because the parser is unable to parser ions and the generated formula is incorrect
  - One of the formulas is manually identified as invalid
- Datapoints are corrected when:
  - There are "typos" (mostly text recognition errors in old papers) in the formulas. Common mistakes include:
    - o/O -> 0 (where Oxygen as confused with zero)
    - capital I, lowercase l, and number 1 confusion
  - Some battery components are not included in the formulas due to the ChemDataExtractor not recognising '–' (dash) as a separator.
  - One of the formulas is manually identified as errorneous when compared to the source text

1.3 3. Using the Dataset

1.3.1 3.1 Selecting the datapoints

Select datapoints with desired battery properties by filtering the Property column and the Specifier column.

Property	Count
Capacity	137532
Voltage	103346
Coulombic Efficiency	10429
Energy	8525
Conductivity	6635

Further information about Capacity is also one-hot encoded into the dataset as features that begin with Capacity_is_ .

Capacity Specifier	Count
Capacity_is_discharge	36600
Capacity_is_charge	8822
Capacity_is_reversible	16703
Capacity_is_specific	13474
Capacity_is_theoretical	6261

1.3.2 3.2 Data cleaning based on warnings

There are 3 basic types of warnings: Serial, Related, and near-Limit. They can be combined to form complex warnings. Please read the original paper for details.

Warning	Count
N/A	113963
S	94469
L	31070
LS	16332
R	7519
RL	3114

1.3.3 3.3 Data cleaning based on the correctness of formulas

Although we have tried our best to identify and correct the errors in the formulas, we probably couldn't find all of them. Formulas might have missing components due to the ChemDataExtractor's sub-optimal ability to deal with separators. Some datapoints might not be valid as the Name_source is not an actual chemical, but some random text strings that the ChemDataExtractor recognised as 'formulas'. In this example 0.7 volts has been confused by the parser as a formula with less than one oxygen atomsand one vanadium atom):

datapoint 2413	
Formulas	['O0.7V']
Name_extracted	[{'O': '0.7', 'V': '1.0'}]
Name_source	O.70V

Sometimes, the ChemDataExtractor is unable to recognise certain acronyms/abbreviations. These acronyms were either ignored or wrongly regarded as regular formulas.

As mentioned in section 2, battery components might be missing due to the ChemDataExtractor's inability to recognise dashes as a separator for heterojunctions. We were able to correct 3569 outof 5974 formulas that might contain heterojunctions, but the remaining 2405 datapoints still have missing components. We recommend that these be dropped.

heterojunction	heterojunction_unresolved	Count
0	0	260493
1	0	3569
1	1	2405

Finally, we recomed you validate formulas against Name_extracted if feasible.

1.3.4 3.4 Formula encoding

This is the most important step before machine learning. Given a list of chemical formulas (as strings), how would you encode it in a ML-compatible way?

We have prepared a periodic table for atom-wise mapping, but in the end the choice is yours.

2 The Code

All changes are reflected in the code except for a few manual corrections recorded in a csv file.

With the original dataset and the correction file one would be able to reproduce the result (assuming the ase python module has already been installed)

[1]:
```
import pandas as pd
import ast
from multiprocessing import Pool
import numpy as np
from ase.formula import Formula  # used as formula validator for heterojunctions
```

2.1 Step 1 - Data Formatting

To format the data so that it is ML friendly and ready for preprocessing. Including: - the deletion of useless columns - the expansion and/or merging of certain columns (e.g. from property type column + property value column to multiple property-value columns) - reindexation so that related datapoints are adjacent - generating the string representation of the formulas, raising formula

warning flags in the process. - merging equivalent specifiers (e.g. capitalised and uncapitalised words)

read files, extract features

[2]:
```
df = pd.read_csv('battery.csv', low_memory = False)
df = df.sort_values(by = ['DOI', 'Name', 'Property', 'Value'])
df.reset_index(drop = True, inplace = True)
```

[3]:
```
def to_number(s):
    try:
        f = float(s)
        return f
    except ValueError:
        return s

# transform

features_kept = [c for c in df if c not in [ 'Value', 'Unit', 'Raw_value',␣
 ↪'Raw_unit']]
def extract_features(dp):
    local_dict = dp[features_kept].to_dict()
    if dp['Property'] == 'Capacity':
        local_dict[dp['Property'] + '/'+ 'Gram^(-1.0)  Hour^(1.0) ␣
 ↪MilliAmpere^(1.0)'.replace(' ', '_')] = dp['Value']
        if dp['Info'] != 'None':
            info = ast.literal_eval(dp['Info'])
            if 'cycle_value' in info.keys():
                local_dict['Cycles'] = info['cycle_value']
            if 'current_units' in info.keys():
                if info['current_units'] in {'C', 'c'}:
                    local_dict['Current/C'] = info['current_value']
                elif info['current_units'] in {'Ag-1','A/g', 'Ag-1'}:
                    eval_ = to_number(info['current_value'])
                    if type(eval_) == float:
                        local_dict['Current/mAg^(-1)'] =  eval_ * 1000
                    else:
                        local_dict['Current/mAg^(-1)'] =  eval_ + '*1000'
                else:
                    local_dict['Current/mAg^(-1)'] = info['current_value']
    else:
```

```
        local_dict[dp['Property'] + '/'+ dp['Unit'].replace(' ', '_')] =␣
 ↪dp['Value']
    return local_dict

with Pool(12) as p:
    result = p.map (extract_features, [row[1] for row in df.iterrows()])

battery = pd.DataFrame(result)
```

[4]:
```
battery.drop(index = df[df['Tag'] == 'Calculated'].index, inplace = True)
battery.drop(columns = ['Correctness', 'Journal', 'Title','Tag', 'Info'],␣
 ↪inplace = True)
battery['Extracted_name'] = battery['Extracted_name'].apply(ast.literal_eval)
```

get formulas, raise warnings

[5]:
```
# deal with a single formula, raise irregular flag if coefficients aren't␣
 ↪numbers
def get_formula(d):
    acc = []
    ireg_flag = 0
    coef_flag = 0
    for k, v in d.items():
        try:
            e = float(v) # cast to float, handel error
            e = int(e) if e.is_integer() else e # cast to int if applicable
            if e >= 100 or e%10 == 0:
                coef_flag = 1
            acc.append(k + (str(e) if e!= 1 else ''))
        except ValueError:
            ireg_flag = 1
            acc.append(k + v)
    return ''.join(acc), ireg_flag, coef_flag

# deal with multiple formula/extracted name, raise flag if irregular␣
 ↪compositions
def get_name(l):

    result = list(map(get_formula, l))
```

```
    return [x[0] for x in result], int(any([x[1] for x in result])),␣
 ↪int(any([x[2] for x in result]))

names = battery['Extracted_name'].apply(get_name)
battery['Formulas'] = names.apply(lambda x: x[0])
battery['Formulas_nonnumeric'] = names.apply(lambda x: x[1])
battery['Formulas_coefficient_warning'] = names.apply(lambda x: x[2])
```

[6]:
```
battery['Formulas_coefficient_warning'].value_counts()
```

[6]:
```
0    281574
1      4021
Name: Formulas_coefficient_warning, dtype: int64
```

[7]:
```
battery['Formulas_nonnumeric'].value_counts()
```

[7]:
```
0    271053
1     14542
Name: Formulas_nonnumeric, dtype: int64
```

feature re-ordering, process specifier values

[8]:
```
order = ['Formulas', 'Formulas_nonnumeric', 'Formulas_coefficient_warning'
         ,'Extracted_name', 'Name','DOI', 'Date'
         ,'Warning', 'Type', 'Property', 'Specifier'
         ,'Capacity/Gram^(-1.0)__Hour^(1.0)__MilliAmpere^(1.0)'
         ,'Cycles','Current/C','Current/mAg^(-1)', 'Voltage/Volt^(1.0)'
         ,'Coulombic Efficiency/Percent^(1.0)'
         ,'Conductivity/CentiMeter^(-1.0)__Siemens^(1.0)'
         ,'Energy/KiloGram^(-1.0)__WattHour^(1.0)']
assert(len(order) == len(battery.columns))
battery = battery[order]
battery['Type'] = battery['Type'].replace('None', np.nan)

battery['Specifier'] = battery['Specifier'].apply(str.lower)
```

```
[9]: merge_dict = {'discharge capacities' : 'discharge capacity',
                'capacities' : 'capacity',
                'reversible capacities' : 'reversible capacity',
                'specific capacities' : 'specific capacity',
                'charge capacities' : 'charge capacity',
                'discharge specific capacities' : 'discharge specific capacity',
                'charge and discharge capacities' : 'discharge and charge␣
 ↪capacities',
                'voltages' : 'voltage',
                'reversible discharge capacities' : 'reversible discharge␣
 ↪capacity',
                'theoretical capacities' : 'theoretical capacity',
                'discharge and charge capacity' : 'discharge and charge␣
 ↪capacities',
                'reversible specific capacities' : 'reversible specific capacity',
                'charge and discharge capacity' : 'discharge and charge␣
 ↪capacities',
                'conductivities' : 'conductivity',
                'reversible charge capacities' : 'reversible charge capacity',
                'charge specific capacities' : 'charge specific capacity',
                'charge and discharge specific capacities' : 'discharge and␣
 ↪charge specific capacities',
                'energies' : 'energy',
                'discharge and charge specific capacity': 'discharge and charge␣
 ↪specific capacities',
                'reversible discharge specific capacities' : 'reversible␣
 ↪discharge specific capacity',
                'electrical conductivities' : 'electrical conductivity',
                'reversible charge specific capacities' : 'reversible charge␣
 ↪specific capacity',
                'charge and discharge specific capacity' : 'discharge and charge␣
 ↪specific capacities',
                'electronic conductivities' : 'electronic conductivity',
                'reversible charge and discharge capacity' : 'reversible␣
 ↪discharge and charge capacities',
                'reversible theoretical capacities' : 'reversible theoretical␣
 ↪capacity',
                'coulombic efficiencies' : 'coulombic effciency'

             }

battery['Specifier'] = battery['Specifier'].apply(lambda x : merge_dict[x] if x␣
 ↪in merge_dict.keys() else x)
battery['Specifier'] = battery.Specifier.replace('none', np.nan)
battery.rename(columns = {'Current/C' : 'Current/C_rate'}, inplace = True)
```

2.2 Step 2 – Finer Feature Processing

including: - one hot encoding of capacity-related Specifiers - extract numeric current values and add as few features

one hot encoding

```
[10]: for spec in ['discharge', 'charge', 'reversible', 'specific', 'theoretical']:
          battery['Capacity_is_'+ spec] = battery['Specifier'].apply(lambda x : 0 if␣
       ↪type(x) != str else (1 if spec in x.split() else 0))
```

dealing with current

```
[11]: cs  = battery[[c for c in battery.columns if c[:7] == 'Current']]
```

```
[12]: c_rate = cs['Current/C_rate'].apply(lambda x : to_number(x) if pd.notna(x) else␣
       ↪x)
      c_rate[pd.notna(c_rate)].apply(type).value_counts()
```

```
[12]: <class 'float'>    13209
      <class 'str'>       4106
      Name: Current/C_rate, dtype: int64
```

```
[13]: c_a = cs['Current/mAg^(-1)'].apply(lambda x : to_number(x) if pd.notna(x) else␣
       ↪x)
      c_a[pd.notna(c_a)].apply(type).value_counts()
```

```
[13]: <class 'float'>    14098
      <class 'str'>       7915
      Name: Current/mAg^(-1), dtype: int64
```

```
[14]: battery.rename(columns = {'Current/C_rate' : 'Current_original/C_rate',
                                'Current/mAg^(-1)' : 'Current_original/mAg^(-1)'},␣
       ↪inplace = True)

      battery['Current/mAg^(-1)'] = c_a.apply(lambda x: np.nan if type(x) == str else␣
       ↪x )
      battery['Current/C_rate'] = c_rate.apply(lambda x: np.nan if type(x) == str␣
       ↪else x )
```

[15]:
```
order = ['Formulas', 'Formulas_nonnumeric', 'Formulas_coefficient_warning'
         ,'Extracted_name', 'Name','DOI', 'Date'
         ,'Warning', 'Type', 'Property', 'Specifier'
         ,'Capacity_is_discharge', 'Capacity_is_charge',␣
 ↪'Capacity_is_reversible'
         , 'Capacity_is_specific', 'Capacity_is_theoretical'
         ,'Capacity/Gram^(-1.0)__Hour^(1.0)__MilliAmpere^(1.0)'
         ,'Cycles', 'Current/C_rate', 'Current_original/C_rate', 'Current/
 ↪mAg^(-1)'
         ,'Current_original/mAg^(-1)' , 'Voltage/Volt^(1.0)'
         ,'Coulombic Efficiency/Percent^(1.0)'
         ,'Conductivity/CentiMeter^(-1.0)__Siemens^(1.0)'
         ,'Energy/KiloGram^(-1.0)__WattHour^(1.0)']
assert(len(order) == len(battery.columns))
battery = battery[order]
```

2.3 Step 3 – Dealing with Errorneous Formulas

including: - manual corrections that focus on datapoints with formula coefficient warning (potential O-> 0 confusion, where Oxygen is encoded as a zero) - dropping datapoints whose formulas have non-numeric coefficients - correction of errorneous patterns - drop datapoints that are ions as the ChemDataExtractor was unable to give correct formulas - restore missing components for some of the datapoints that have dashes (–) in its original name / name recorded by the original paper

**merge corrections**

[16]:
```
corrections = pd.read_csv('battery_corrected.csv', low_memory=False)
```

[17]:
```
battery['Formulas'] = corrections['Formulas']
battery['Formulas_nonnumeric'] = corrections['Formulas_nonnumeric']
```

[18]:
```
battery['Formulas'] = battery['Formulas'].apply(ast.literal_eval)
# battery['Extracted_name'] = battery['Extracted_name'].apply(ast.literal_eval)
```

**drop datapoints with non-numeric coefficients**

[19]:
```python
# drop non- numeric coefficients
battery.drop (index = battery[battery.Formulas_nonnumeric ==1].index, inplace = True
)
```

pattern matching errors

[20]:
```python
def find_datapoints_with_substrings(df, strings):
    return df[df.Formulas.apply(lambda x: any([any([typo in elem for typo in strings]) for elem in x]))]

def replace_substrings(df, old, new):
    return df.Formulas.apply(lambda x: [elem.replace(old, new) for elem in x])
```

[21]:
```python
# correcting O
battery['Formulas'] = replace_substrings(battery, 'C1O4', 'ClO4')
battery['Formulas'] = replace_substrings(battery, 'Hg0', 'HgO')
battery['Formulas'] = replace_substrings(battery, 'Si0O', 'SiO')
battery['Formulas'] = replace_substrings(battery, 'SiOP', 'SiP')
battery['Formulas'] = replace_substrings(battery, 'CIO4', 'ClO4')
battery['Formulas'] = replace_substrings(battery, 'CIO', 'ClO')
```

dropping ions

[22]:
```python
# drop ions according to Name
ions_1_indies = battery[battery.Name.apply(lambda x: any([c in x for c in ['(II)', '(III)', '(I)', '(IV)', '(V)', '(VI)', '(VII)']]))].index
ions_2_indies = battery[battery.Name.apply(lambda x: any([c in x for c in ['+ /']]))].index
battery.drop (index = ions_1_indies, inplace=True)
battery.drop (index = ions_2_indies, inplace=True)
```

[23]:
```python
# drop CO - misc error
idx = battery[battery.Formulas.apply(lambda x: any([ elem == 'CO' for elem in x]))].index
battery.drop(index = idx, inplace = True)
```

[24]:
```python
battery.drop(columns = ['Formulas_nonnumeric', 'Formulas_coefficient_warning'], inplace=True)
battery.rename(columns={'Name' : 'Name_source', 'Extracted_name' : 'Name_extracted'}, inplace = True)
```

[25]:
```
battery['heterojunction'] = battery.Name_source.apply(lambda x: 1 if any([c in␣
↪x for c in ['-']]) else 0)
order = list(battery.columns)[:3] + ['heterojunction'] + list(battery.
↪columns)[3:len(battery.columns)-1]
battery = battery[order]
hjs = battery[battery.heterojunction == 1][['Formulas', 'Name_source']]
```

[26]:
```
# see what simple splits can do

hjs['split'] = hjs.Name_source.apply(lambda x : [elem.strip() for elem in x.
↪split('-')])

def check_split(split):
    is_valid = []
    for string in split:
        try:
            f = Formula(string, strict = True)
            is_valid.append(True)
        except ValueError:
            is_valid.append(False)
    return is_valid

hjs['valid_formulas'] = hjs.split.apply(check_split)
hjs['valid_split'] = hjs.valid_formulas.apply(all)
```

[27]:
```
# 2730 instances corrected
hjs.valid_split.value_counts()
```

[27]:
```
False    3244
True     2730
Name: valid_split, dtype: int64
```

[28]:
```
# correct the instances in the original dataframe

corrected = battery.apply(lambda x : hjs['split'][x.name]
                          if (x.heterojunction == 1) and
                          (hjs['valid_split'][x.name] == True) else x.Formulas,␣
↪axis = 1)
battery['Formulas'] = corrected
```

[29]:
```
# for uncorrected instances
hjs = hjs[hjs.valid_split == False]
# split with @, // ,/
for sep in ['@', '//', '/']:
    new = hjs.split.apply(lambda x : [val.strip() for sublist in [elem.
↪split(sep) for elem in x] for val in sublist])
    hjs['split'] = new
```

```
hjs['valid_formulas'] = hjs.split.apply(check_split)
hjs['valid_split'] = hjs.valid_formulas.apply(all)
```

[30]:
```
# 839 instances corrected
hjs.valid_split.value_counts()
```

[30]:
```
False    2405
True      839
Name: valid_split, dtype: int64
```

[31]:
```
# correrct formulas for CNTs (Carbon Nanotubes)
hjs['split'] = hjs.split.apply(lambda x : ['C' if f == 'CNTs' else f for f in␣
 ↪x])
```

[32]:
```
# correct the instances in the original dataframe

corrected = battery.apply(lambda x : hjs['split'][x.name]
                          if (x.name in hjs.index) and
                          (hjs['valid_split'][x.name] == True) else x.Formulas,␣
 ↪axis = 1)
battery['Formulas'] = corrected
```

[33]:
```
# mark unresolved instances in the original dataframe
hjs = hjs[hjs.valid_split == False]
battery['heterojunction_unresolved'] = battery.apply(lambda x : 1 if x.name in␣
 ↪hjs.index else 0,axis = 1)
```

[34]:
```
order = list(battery.columns[:4]) + list(battery.columns[-1:] )+ list(battery.
 ↪columns[4:-1])
battery = battery[order]
```

[35]:
```
battery.heterojunction_unresolved.value_counts()
```

[35]:
```
0    264062
1      2405
Name: heterojunction_unresolved, dtype: int64
```

[36]:
```
battery.heterojunction.value_counts()
```

[36]:
```
0    260493
1      5974
Name: heterojunction, dtype: int64
```

[37]:
```
battery['Warning'] = battery.Warning.replace ('None', np.nan)
```

[ ]:
```
battery.describe()
```

periodic_table.csv

num	symbol	number	row	group	atomic_m	molar_vol	atomic_ra	ionic_radi	covalent_r	van_der_v	electron_r	electron_a	electrical_	thermal_c	boiling_po	melting_p	Tc	enthalpy_	first_ioniz	second_io
0	H	1	1	1	1.00794	11.42	1.008	0	0.32	120	2.2	72.769	0	0.1805	20.28	14.01	33	0.558	1312	1312
1	He	2	1	18	4.002602	21	1.4	0	0.37	140	4.16	0	0	0.1513	4.22	0.95	5.19	0.02	2372	5250
2	Li	3	2	1	6.941	13.02	1.82	0.9	1.3	212	0.98	59.633	9.50E-08	85	1615	453.69	3223	3	520	7298
3	Be	4	2	2	9.012182	4.85	1.53	0.59	0.99	198	1.57	0	3.80E-08	190	2742	1560	0	7.95	899.5	1757.1
4	B	5	2	13	10.811	4.39	1.92	0.41	0.84	192	2.04	26.989	1.00E+12	27	4200	2349	0	50	800.6	2427.1
5	C	6	2	14	12.0107	5.29	1.7	0.3	0.75	177	2.55	121.776	1.00E-05	140	4300	3800	0	117	1086	2352
6	N	7	2	15	14.0067	13.54	1.55	0.63	0.71	166	3.04	0	0	0.02583	77.36	63.05	126.2	0.36	1402	2856
7	O	8	2	16	15.9994	17.36	1.52	1.26	0.64	150	3.44	140.976	0	0.02658	90.2	54.8	154.6	0.222	1313	3388
8	F	9	2	17	18.9984	11.2	1.47	0.705	0.6	146	3.98	328.165	0	0.0277	85.03	53.53	144	0.26	1681	3374
9	Ne	10	2	18	20.1797	13.23	1.54	0	0.62	158	4.79	0	0	0.0491	27.07	24.56	44.4	0.34	2080	3952
10	Na	11	3	1	22.98977	23.78	2.27	1.16	1.6	250	0.93	52.867	4.90E-08	140	1156	370.87	2573	2.6	495.8	4562
11	Mg	12	3	2	24.305	14	1.73	0.86	1.4	251	1.31	0	4.40E-08	160	1363	923	0	8.7	737.7	1450.7
12	Al	13	3	13	26.98154	10	1.84	0.675	1.24	225	1.61	41.762	2.70E-08	235	2792	933.47	0	10.7	577.5	1816.7
13	Si	14	3	14	28.0855	12.06	2.1	0.54	1.14	219	1.98	133.068	0.001	150	3173	1687	0	50.2	786.5	1577.1
14	P	15	3	15	30.97376	17.02	1.8	0.55	1.09	190	2.19	72.037	1.00E-07	0.236	550	317	994	0.64	1011	1907
15	S	16	3	16	32.065	15.53	1.8	0.88	1.04	189	2.58	200.41	1.00E+23	0.205	717.87	388.36	1314	1.73	999.6	2252
16	Cl	17	3	17	35.453	17.39	1.75	0.78	1	182	3.16	348.575	1E+10	0.0089	239.11	171.6	417	3.2	1251.2	2298
17	Ar	18	3	18	39.948	22.56	1.88	0	1.01	183	3.24	0	0	0.01772	87.3	83.8	150.8	1.18	1520	2665.8
18	K	19	4	1	39.0983	45.94	2.75	1.52	2	273	0.82	48.385	7.50E-08	100	1032	336.53	2223	2.33	418.8	3052
19	Ca	20	4	2	40.078	26.2	2.31	1.14	1.74	262	1.01	2.37	3.40E-08	200	1757	1115	0	8.54	589.8	1145.4
20	Sc	21	4	3	44.95591	15	2.15	0.885	1.59	258	1.36	18.1	5.50E-07	16	3103	1814	0	16	633	1235
21	Ti	22	4	4	47.867	10.64	2.11	0.851667	1.48	246	1.54	7.6	4.00E-07	22	3560	1941	0	18.7	658.8	1309
22	V	23	4	5	50.9415	8.32	2.07	0.7775	1.44	242	1.63	50.6	2.00E-07	31	3680	2183	0	22.8	650.9	1414
23	Cr	24	4	6	51.9961	7.23	2.06	0.94	1.3	245	1.66	64.3	1.27E-07	94	2944	2180	0	20.5	652.9	1590.6
24	Mn	25	4	7	54.93805	7.35	2.05	0.648333	1.29	245	1.55	0	1.44E-06	7.8	2334	1519	0	13.2	717.3	1509
25	Fe	26	4	8	55.845	7.09	2.04	0.8525	1.24	244	1.83	14.569	1.00E-07	80	3134	1811	0	13.8	762.5	1561.9
26	Co	27	4	9	58.9332	6.67	2	0.768333	1.18	240	1.88	63.873	6.00E-08	100	3200	1768	0	16.2	760.4	1648
27	Ni	28	4	10	58.6934	6.59	1.97	0.74	1.17	240	1.92	111.537	7.20E-08	91	3186	1728	0	17.2	737.1	1753
28	Cu	29	4	11	63.546	7.11	1.96	0.82	1.22	238	1.9	119.159	1.72E-08	400	3200	1357.77	0	13.1	745.5	1957.9
29	Zn	30	4	12	65.409	9.16	2.01	0.88	1.2	239	1.65	0	6.00E-08	120	1180	692.68	0	7.35	906.4	1733.3
30	Ga	31	4	13	69.723	11.8	1.87	0.76	1.23	232	1.81	41.49	1.40E-07	29	2477	302.91	0	5.59	578.8	1979.3
31	Ge	32	4	14	72.64	13.63	2.11	0.77	1.2	229	2.01	118.939	0.0005	60	3093	1211.4	0	31.8	762	1537.5
32	As	33	4	15	74.9216	12.95	1.85	0.66	1.2	188	2.18	77.57	3.30E-07	50	887	1090	1700	27.7	947	1798
33	Se	34	4	16	78.96	16.42	1.9	1.013333	1.18	182	2.55	194.965	1.00E-08	0.52	958	494	1766	5.4	941	2045
34	Br	35	4	17	79.904	19.78	1.85	0.8825	1.17	186	2.96	324.537	1.00E+18	0.12	332	265.8	586	5.8	1139.9	2103
35	Kr	36	4	18	83.798	27.99	2.02	0	1.16	225	3	0	0	0.00943	119.93	115.79	209.4	1.64	1350.8	2350.4
36	Rb	37	5	1	85.4678	55.76	3.03	1.66	2.15	321	0.82	46.884	1.33E-07	58	961	312.46	2093	2.19	403	2633
37	Sr	38	5	2	87.62	33.94	2.49	1.32	1.9	284	0.95	4.631	1.35E-07	35	1655	1050	0	8	549.5	1064.2
38	Y	39	5	3	88.90585	19.88	2.32	1.04	1.76	275	1.22	29.621	6.00E-07	17	3609	1799	0	11.4	600	1180
39	Zr	40	5	4	91.224	14.02	2.23	0.86	1.64	252	1.33	41.103	4.33E-07	23	4682	2128	0	21	640	1270
40	Nb	41	5	5	92.90638	10.83	2.18	0.82	1.56	256	1.59	88.381	1.52E-07	54	5017	2750	0	26.8	652.1	1380
41	Mo	42	5	6	95.94	9.38	2.17	0.775	1.46	245	2.16	72.171	5.50E-08	139	4912	2896	0	36	684.3	1560
42	Tc	43	5	7	98	8.63	2.16	0.741667	1.38	244	1.91	53.07	2.20E-07	51	4538	2430	0	23	702	1470
43	Ru	44	5	8	101.07	8.17	2.13	0.661	1.36	246	2.2	101.31	7.10E-08	120	4423	2607	0	25.7	710.2	1620
44	Rh	45	5	9	102.9055	8.28	2.1	0.745	1.34	244	2.28	109.704	4.30E-08	150	3968	2237	0	21.7	719.7	1740
45	Pd	46	5	10	106.42	8.56	2.1	0.84625	1.3	215	2.2	53.225	1.08E-07	72	3236	1828.05	0	16.7	804.4	1870
46	Ag	47	5	11	107.8682	10.27	2.11	1.086667	1.36	253	1.93	125.624	1.63E-08	430	2435	1234.93	0	11.3	731	2070
47	Cd	48	5	12	112.411	13	2.18	1.09	1.4	249	1.69	0	7.00E-08	97	1040	594.22	0	6.3	867.8	1631.4
48	In	49	5	13	114.818	15.76	1.93	0.94	1.42	243	1.78	28.9	8.00E-08	82	2345	429.75	0	3.26	558.3	1820.7
49	Sn	50	5	14	118.71	16.29	2.17	0.83	1.4	242	1.96	107.298	1.15E-07	67	2875	505.08	0	7	708.6	1411.8
50	Sb	51	5	15	121.76	18.19	2.06	0.83	1.4	247	2.05	100.924	4.00E-07	24	1860	903.78	0	19.7	834	1594.9
51	Te	52	5	16	127.6	20.46	2.06	1.293333	1.37	199	2.12	190.161	0.0001	3	1261	722.66	0	17.5	869.3	1790
52	I	53	5	17	126.9045	25.72	1.98	1.273333	1.36	204	2.66	295.152	1.00E+15	0.449	457.4	386.85	819	7.76	1008.4	1845.9
53	Xe	54	5	18	131.293	35.92	2.16	0.62	1.36	206	2.6	0	0	0.00565	165.1	161.4	289.7	2.3	1170.4	2046.4
54	Cs	55	6	1	132.9055	70.94	3.43	1.81	2.38	348	0.79	45.505	2.10E-07	36	944	301.59	1938	2.09	375.7	2234.3
55	Ba	56	6	2	137.327	38.16	2.68	1.49	2.06	303	0.89	13.954	3.40E-07	18	2143	1000	0	8	502.9	965.2
56	La	57	8	3	138.9055	22.39	2.43	1.172	1.94	298	1.1	45.35	6.15E-07	13	3743	1193	0	6.2	538.1	1067
57	Ce	58	8	4	140.116	20.69	2.42	1.08	1.84	288	1.12	62.72	7.40E-07	11	3633	1068	0	5.5	534.4	1050
58	Pr	59	8	5	140.9077	20.8	2.4	1.06	1.9	292	1.13	92.819	7.00E-07	13	3563	1208	0	6.9	527	1020
59	Nd	60	8	6	144.242	20.59	2.39	1.2765	1.88	295	1.14	0	6.43E-07	17	3373	1297	0	7.1	533.1	1040
60	Pm	61	8	7	145	20.23	2.38	1.11	1.86	290	1.13	0	7.50E-07	15	3273	1373	0	7.7	540	1050
61	Sm	62	8	8	150.36	19.98	2.36	1.229	1.85	287	1.17	0	9.40E-07	13	2076	1345	0	8.6	544.5	1070
62	Eu	63	8	9	151.964	28.97	2.35	1.1985	1.83	283	1.19	83.363	9.00E-07	14	1800	1099	0	9.2	547.1	1085
63	Gd	64	8	10	157.25	19.9	2.34	1.075	1.82	279	1.21	0	1.31E-06	11	3523	1585	0	10	593.4	1170
64	Tb	65	8	11	158.9254	19.3	2.33	0.9815	1.81	287	1.13	0	1.15E-06	11	3503	1629	0	10.8	565.8	1110
65	Dy	66	8	12	162.5	19.01	2.31	1.131	1.8	281	1.22	0	9.26E-07	11	2840	1680	0	11.1	573	1130
66	Ho	67	8	13	164.9303	18.74	2.3	1.041	1.79	283	1.23	0	8.14E-07	16	2993	1734	0	17	581	1140
67	Er	68	8	14	167.259	18.46	2.29	1.03	1.77	279	1.24	0	8.60E-07	15	3141	1802	0	19.9	589.3	1150
68	Tm	69	8	15	168.9342	19.1	2.27	1.095	1.77	280	1.25	99.283	6.76E-07	17	2223	1818	0	16.8	596.7	1160
69	Yb	70	8	16	173.04	24.84	2.26	1.084	1.78	274	1.26	-1.93	2.50E-07	39	1469	1097	0	7.7	603.4	1174.8
70	Lu	71	8	17	174.967	17.78	2.24	1.001	1.74	263	1.27	32.81	5.80E-07	16	3675	1925	0	22	523.5	1340
71	Hf	72	6	4	178.49	13.44	2.23	0.85	1.64	253	1.32	1.351	3.40E-07	23	4876	2506	0	25.5	658.5	1440
72	Ta	73	6	5	180.9479	10.85	2.22	0.82	1.58	257	1.51	31.068	1.35E-07	57	5731	3290	0	36	761	1500
73	W	74	6	6	183.84	9.47	2.18	0.766667	1.5	249	2.36	78.757	5.40E-08	170	5828	3695	0	35	770	1700
74	Re	75	6	7	186.207	8.86	2.16	0.7125	1.41	248	1.93	14.47	1.80E-07	48	5869	3459	0	33	760	1260
75	Os	76	6	8	190.23	8.42	2.13	0.673	1.36	241	2.18	106.1	8.10E-08	88	5285	3306	0	31	840	1600
76	Ir	77	6	9	192.217	8.52	2.13	0.765	1.32	229	2.2	150.884	4.70E-08	150	4701	2739	0	26	880	1600
77	Pt	78	6	10	195.084	9.09	2.13	0.805	1.3	232	2.28	205.321	1.06E-07	72	4098	2041.4	0	20	870	1791
78	Au	79	6	11	196.9666	10.21	2.14	1.07	1.3	245	2.54	222.749	2.20E-08	320	3129	1337.33	0	12.5	890	1980
79	Hg	80	6	12	200.59	14.09	2.23	1.245	1.32	247	2	0	9.60E-07	8.3	629.88	234.32	1750	2.29	1007.1	1810
80	Tl	81	6	13	204.3833	17.22	1.96	1.3325	1.44	260	1.62	36.375	1.50E-07	46	1746	577	0	4.2	589.4	1971
81	Pb	82	6	14	207.2	18.26	2.02	1.1225	1.45	254	2.33	35.121	2.10E-07	35	2022	600.61	0	4.77	715.6	1450.5
82	Bi	83	6	15	208.9804	21.31	2.07	1.035	1.5	280	2.02	90.924	1.30E-06	8	1837	544.4	0	10.9	703	1610
83	Po	84	6	16	210	22.97	1.97	0.945	1.42	293	1.99	183.3	4.00E-07	20	1235	527	0	13	812.1	
84	At	85	6	17	210	33	2.02	0.76	1.48	288	2.22	270.2	0	2	575	575	0	6	920	
85	Rn	86	6	18	220	50.5	2.2	0	1.46	271	2.43	0	0	0.00361	211.3	202	377	3	1037	
86	Fr	87	7	1	223	77	3.48	1.94	2.42	282	0.71	44.38	0	15	300	300	0	2	380	
87	Ra	88	7	2	226	41.09	2.83	1.62	2.11	281	0.92	9.65	1.00E-06	19	2010	973	0	8	509.3	979
88	Ac	89	9	3	227	22.55	2.47	1.26	2.01	283	1.09	33.77	0	12	3573	1323	0	14	499	1170
89	Th	90	9	4	232.0381	19.8	2.45	1.08	1.9	305	1.32	0	1.50E-07	54	5093	2115	0	16	587	1110
90	Pa	91	9	5	231.0359	15.18	2.43	1.04	1.84	340	1.54	0	1.80E-07	47	1841	1841	0	15	568	
91	U	92	9	6	238.0289	12.49	2.41	0.99125	1.83	305	1.7	0	2.80E-07	27	4200	1405.3	0	14	597.6	1420
92	Np	93	9	7	237	11.59	2.39	1	1.8	270	1.36	0	1.20E-06	6	4273	910	0	10	604.5	
93	Pu	94	9	8	244	12.29	2.43	0.9675	1.8	0	1.3	0	1.50E-06	6	3503	912.5	0	2.8	584.7	
94	Am	95	9	9	243	17.63	2.44	1.168333	1.73	0	1.3	0	0	10	2880	1449	0	0	578	
95	Cm	96	9	10	247	18.05	2.45	1.05	1.68	0	1.3	0	0	8.8	3383	1613	0	0	581	
96	Bk	97	9	11	247	16.84	2.44	1.035	1.68	0	1.3	0	0	10	1259	1259	0	0	601	
97	Cf	98	9	12	251	16.5	2.45	1.0255	1.68	0	1.3	0	0	10	1173	1173	0	0	608	1160
98	Es	99	9	13	252	28.52	2.45	0	1.65	0	1.3	0	0	10	1133	1133	0	0	619	
99	Fm	100	9	14	257	0	2.45	0	1.67	0	1.3	0	0	10	1800	1800	0	0	627	
100	Md	101	9	15	258	0	2.46	0	1.73	0	1.33	0	0	10	1100	1100	0	0	635	
101	No	102	9	16	259	0	2.46	1.24	1.76	0	1.36	0	0	10	1100	1100	0	0	642	
102	Lr	103	9	17	262	0	2.46	0	1.61	0	1.34	0	0	10	1900	1900	0	0	472.8	

num	symbol	s	p	d	f	Omax	Omin	valance	block	electron_s	metal	noble_gas	alkali_met	rare_earth	Over_met	Lanthanid	actinoid	Halogen	alkaline	poissons_	bulk_mod	youngs_m	rigidity_m	thermal_expansion
0	H	1	0	0	0	1	-1	1	s	1	0	0	0	0	0	0	0	0	0					
1	He	2	0	0	0	0	0	0	s	2	0	1	0	0	0	0	0	0	0					
2	Li	1	0	0	0	1	1	1	s	2.1	1	0	1	0	0	0	0	0	0		11	4.9	4.2	4.60E-05
3	Be	2	0	0	0	2	2	2	s	2.2	1	0	0	0	0	0	0	0	1	0.032	130	287	132	1.13E-05
4	B	2	1	0	0	3	1	3	p	2.3	0	0	0	0	0	0	0	0	0		320			6.00E-06
5	C	2	2	0	0	4	-4	4	p	2.4	0	0	0	0	0	0	0	0	0		33			7.10E-06
6	N	2	3	0	0	5	-3	3	p	2.5	0	0	0	0	0	0	0	0	0					
7	O	2	4	0	0	2	-2	2	p	2.6	0	0	0	0	0	0	0	0	0					
8	F	2	5	0	0	-1	-1	1	p	2.7	0	0	0	0	0	0	0	1	0					
9	Ne	2	6	0	0	0	0	0	p	2.8	0	1	0	0	0	0	0	0	0					
10	Na	1	0	0	0	1	-1	1	s	2.8.1	1	0	1	0	0	0	0	0	0		6.3	10	3.3	7.10E-05
11	Mg	2	0	0	0	2	1	2	s	2.8.2	1	0	0	0	0	0	0	0	1	0.29	45	45	17	8.20E-06
12	Al	2	1	0	0	3	1	3	p	2.8.3	1	0	0	0	0	0	0	0	0	0.35	76	70	26	2.31E-05
13	Si	2	2	0	0	4	-4	4	p	2.8.4	0	0	0	0	0	0	0	0	0		100	47		2.60E-06
14	P	2	3	0	0	5	-3	5	p	2.8.5	0	0	0	0	0	0	0	0	0		11			
15	S	2	4	0	0	6	-2	6	p	2.8.6	0	0	0	0	0	0	0	0	0		7.7			
16	Cl	2	5	0	0	7	-1	5	p	2.8.7	0	0	0	0	0	0	0	1	0		1.1			
17	Ar	2	6	0	0	0	0	0	p	2.8.8	0	1	0	0	0	0	0	0	0					
18	K	1	0	0	0	1	-1	1	s	2.8.8.1	1	0	1	0	0	0	0	0	0		3.1		1.3	
19	Ca	2	0	0	0	2	2	2	s	2.8.8.2	1	0	0	0	0	0	0	0	1	0.31	17	20	7.4	2.23E-05
20	Sc	2	0	1	0	3	1	3	d	2.8.9.2	1	0	0	0	1	0	0	0	0	0.28	57	74	29	1.02E-05
21	Ti	2	0	2	0	4	-1	4	d	2.8.10.2	1	0	0	0	1	0	0	0	0	0.32	110	116	44	8.60E-06
22	V	2	0	3	0	5	-1	5	d	2.8.11.2	1	0	0	0	1	0	0	0	0	0.37	160	128	47	8.40E-06
23	Cr	1	0	5	0	6	-2	6	d	2.8.13.1	1	0	0	0	1	0	0	0	0	0.21	160	279	115	4.90E-06
24	Mn	2	0	5	0	7	-3	4	d	2.8.12.2	1	0	0	0	1	0	0	0	0		120	198		2.17E-05
25	Fe	2	0	6	0	6	-2	3	d	2.8.14.2	1	0	0	0	1	0	0	0	0	0.29	170	211	82	1.18E-05
26	Co	2	0	7	0	5	-1	4	d	2.8.15.2	1	0	0	0	1	0	0	0	0	0.31	180	209	75	1.30E-05
27	Ni	2	0	8	0	4	-1	2	d	2.8.16.2	1	0	0	0	1	0	0	0	0	0.31	180	200	76	1.34E-05
28	Cu	1	0	10	0	4	1	2	d	2.8.18.1	1	0	0	0	1	0	0	0	0	0.34	140	130	48	1.65E-05
29	Zn	2	0	10	0	2	1	2	d	2.8.18.2	1	0	0	0	1	0	0	0	0	0.25	70	108	43	3.02E-05
30	Ga	2	1	10	0	3	1	3	p	2.8.18.3	1	0	0	0	0	0	0	0	0					0.00012
31	Ge	2	2	10	0	4	-4	4	p	2.8.18.4	1	0	0	0	0	0	0	0	0					6.00E-06
32	As	2	3	10	0	5	-3	5	p	2.8.18.5	0	0	0	0	0	0	0	0	0		22	8		
33	Se	2	4	10	0	6	-2	6	p	2.8.18.6	0	0	0	0	0	0	0	0	0	0.33	8.3	10	3.7	
34	Br	2	5	10	0	7	-1	5	p	2.8.18.7	0	0	0	0	0	0	0	1	0		1.9			
35	Kr	2	6	10	0	0	0	2	p	2.8.18.8	0	1	0	0	0	0	0	0	0					
36	Rb	1	0	0	0	1	1	1	s	2.8.18.8.1	1	0	1	0	0	0	0	0	0		2.5	2.4		
37	Sr	2	0	0	0	2	2	2	s	2.8.18.8.2	1	0	0	0	0	0	0	0	1	0.28			6.1	2.25E-05
38	Y	2	0	1	0	3	1	3	d	2.8.18.9.2	1	0	0	0	1	0	0	0	0	0.24	41	64	26	1.06E-05
39	Zr	2	0	2	0	4	1	4	d	2.8.18.10.	1	0	0	0	1	0	0	0	0	0.34		68	33	5.70E-06
40	Nb	1	0	4	0	5	-1	5	d	2.8.18.12.	1	0	0	0	1	0	0	0	0	0.4	170	105	38	7.30E-06
41	Mo	1	0	5	0	6	-2	6	d	2.8.18.13.	1	0	0	0	1	0	0	0	0	0.31	230	329	20	4.80E-06
42	Tc	2	0	5	0	7	-3	6	d	2.8.18.14.	1	0	0	0	1	0	0	0	0					
43	Ru	1	0	7	0	8	-2	6	d	2.8.18.15.	1	0	0	0	1	0	0	0	0	0.3	220	447	173	6.40E-06
44	Rh	1	0	8	0	6	-1	6	d	2.8.18.16.	1	0	0	0	1	0	0	0	0	0.26	380	275	150	8.20E-06
45	Pd	0	0	10	0	4	2	4	d	2.8.18.18.	1	0	0	0	1	0	0	0	0	0.39	180	121	44	1.18E-05
46	Ag	1	0	10	0	3	1	1	d	2.8.18.18.	1	0	0	0	1	0	0	0	0	0.37	100	83	30	1.89E-05
47	Cd	2	0	10	0	2	1	2	d	2.8.18.18.	1	0	0	0	1	0	0	0	0	0.3	42	50	19	3.08E-05
48	In	2	1	10	0	3	1	3	p	2.8.18.18.	1	0	0	0	0	0	0	0	0			11		3.21E-05
49	Sn	2	2	10	0	4	-4	4	p	2.8.18.18.	1	0	0	0	0	0	0	0	0	0.36	58	50	18	2.20E-05
50	Sb	2	3	10	0	5	-3	5	p	2.8.18.18.	1	0	0	0	0	0	0	0	0		42	55	20	1.10E-05
51	Te	2	4	10	0	6	-2	6	p	2.8.18.18.	0	0	0	0	0	0	0	0	0		65	43	16	
52	I	2	5	10	0	7	-1	7	p	2.8.18.18.	0	0	0	0	0	0	0	1	0		7.7			
53	Xe	2	6	10	0	0	0	6	p	2.8.18.18.	0	1	0	0	0	0	0	0	0					
54	Cs	1	0	0	0	1	1	1	s	2.8.18.18.	1	0	1	0	0	0	0	0	0		1.6	1.7		
55	Ba	2	0	0	0	2	2	2	s	2.8.18.18.	1	0	0	0	0	0	0	0	1		9.6	13	4.9	2.06E-05
56	La	2	0	1	0	3	2	3	f	2.8.18.18.	1	0	0	1	1	1	0	0	0	0.28	28	37	14	1.21E-05
57	Ce	2	0	1	1	4	2	4	f	2.8.18.19.	1	0	0	1	1	1	0	0	0	0.24	22	34	14	6.30E-06
58	Pr	2	0	0	3	4	2	4	f	2.8.18.21.	1	0	0	1	1	1	0	0	0	0.28	29	37	15	6.70E-06
59	Nd	2	0	0	4	3	2	3	f	2.8.18.22.	1	0	0	1	1	1	0	0	0	0.28	32	41	16	9.60E-06
60	Pm	2	0	0	5	3	3	3	f	2.8.18.23.	1	0	0	1	1	1	0	0	0	0.28	33	46	18	1.10E-05
61	Sm	2	0	0	6	3	2	3	f	2.8.18.24.	1	0	0	1	1	1	0	0	0	0.27	38	50	20	1.27E-05
62	Eu	2	0	0	7	3	2	3	f	2.8.18.25.	1	0	0	1	1	1	0	0	0	0.15	8.3	18	7.9	3.50E-05
63	Gd	2	0	1	7	3	1	3	f	2.8.18.25.	1	0	0	1	1	1	0	0	0	0.26	38	55	22	9.40E-06
64	Tb	2	0	0	9	4	1	3	f	2.8.18.27.	1	0	0	1	1	1	0	0	0	0.26	38.7	56	22	1.03E-05
65	Dy	2	0	0	10	3	2	3	f	2.8.18.28.	1	0	0	1	1	1	0	0	0	0.25	41	61	25	9.90E-06
66	Ho	2	0	0	11	3	3	3	f	2.8.18.29.	1	0	0	1	1	1	0	0	0	0.23	40	65	26	1.12E-05
67	Er	2	0	0	12	3	3	3	f	2.8.18.30.	1	0	0	1	1	1	0	0	0	0.24	44	70	28	1.22E-05
68	Tm	2	0	0	13	3	2	3	f	2.8.18.31.	1	0	0	1	1	1	0	0	0	0.21	45	74	31	1.33E-05
69	Yb	2	0	0	14	3	2	3	f	2.8.18.32.	1	0	0	1	1	1	0	0	0	0.21	31	24	9.9	2.63E-05
70	Lu	2	0	1	14	3	3	3	d	2.8.18.32.	1	0	0	1	1	1	0	0	0	0.26	48	69	27	9.90E-06
71	Hf	2	0	2	14	4	2	4	d	2.8.18.32.	1	0	0	0	1	0	0	0	0	0.37	110	78	30	5.90E-06
72	Ta	2	0	3	14	5	-1	5	d	2.8.18.32.	1	0	0	0	1	0	0	0	0	0.34	200	186	69	6.30E-06
73	W	2	0	4	14	6	-2	6	d	2.8.18.32.	1	0	0	0	1	0	0	0	0	0.28	310	411	161	4.50E-06
74	Re	2	0	5	14	7	-3	7	d	2.8.18.32.	1	0	0	0	1	0	0	0	0	0.3	370	463	178	6.20E-06
75	Os	2	0	6	14	8	-2	6	d	2.8.18.32.	1	0	0	0	1	0	0	0	0	0.25			222	5.10E-06
76	Ir	2	0	7	14	6	-3	6	d	2.8.18.32.	1	0	0	0	1	0	0	0	0	0.26	320	528	210	6.40E-06
77	Pt	1	0	9	14	6	-2	6	d	2.8.18.32.	1	0	0	0	1	0	0	0	0	0.38	230	168	61	8.80E-06
78	Au	1	0	10	14	5	-1	5	d	2.8.18.32.	1	0	0	0	1	0	0	0	0	0.44	220	78	27	1.42E-05
79	Hg	2	0	10	14	4	1	2	d	2.8.18.32.	1	0	0	0	1	0	0	0	0		25			
80	Tl	2	1	10	14	3	1	3	p	2.8.18.32.	1	0	0	0	0	0	0	0	0	0.45	43	8	2.8	2.99E-05
81	Pb	2	2	10	14	4	-4	4	p	2.8.18.32.	1	0	0	0	0	0	0	0	0	0.44	46	16	5.6	2.89E-05
82	Bi	2	3	10	14	5	-3	5	p	2.8.18.32.	1	0	0	0	0	0	0	0	0	0.33	31	32	12	1.34E-05
83	Po	2	4	10	14	6	-2	6	p	2.8.18.32.	1	0	0	0	0	0	0	0	0					
84	At	2	5	10	14	5	-1	7	p	2.8.18.32.	0	0	0	0	0	0	0	1	0					
85	Rn	2	6	10	14	0	0	6	p	2.8.18.32.	0	1	0	0	0	0	0	0	0					
86	Fr	1	0	0	0	1	1	1	s	2.8.18.32.	1	0	1	0	0	0	0	0	0					
87	Ra	2	0	0	0	2	2	2	s	2.8.18.32.	1	0	0	0	0	0	0	0	1					
88	Ac	2	0	1	0	3	3	3	f	2.8.18.32.	1	0	0	1	1	0	1	0	0					
89	Th	2	0	2	0	4	2	4	f	2.8.18.32.	1	0	0	1	1	0	1	0	0	0.27	54	79	31	1.10E-05
90	Pa	2	0	1	2	5	3	5	f	2.8.18.32.	1	0	0	1	1	0	1	0	0					
91	U	2	0	1	3	6	3	6	f	2.8.18.32.	1	0	0	1	1	0	1	0	0	0.23	100	208	111	1.39E-05
92	Np	2	0	1	4	7	3	6	f	2.8.18.32.	1	0	0	1	1	0	1	0	0					
93	Pu	2	0	0	6	7	3	6	f	2.8.18.32.	1	0	0	1	1	0	1	0	0	0.21		96	43	
94	Am	2	0	0	7	6	2	4	f	2.8.18.32.	1	0	0	1	1	0	1	0	0					
95	Cm	2	0	1	7	4	3	4	f	2.8.18.32.	1	0	0	1	1	0	1	0	0					
96	Bk	2	0	0	9	4	3	4	f	2.8.18.32.	1	0	0	1	1	0	1	0	0					
97	Cf	2	0	0	10	4	2	4	f	2.8.18.32.	1	0	0	1	1	0	1	0	0					
98	Es	2	0	0	11	3	2	4	f	2.8.18.32.	1	0	0	1	1	0	1	0	0					
99	Fm	2	0	0	12	3	2	3	f	2.8.18.32.	1	0	0	1	1	0	1	0	0					
100	Md	2	0	0	13	3	2	3	f	2.8.18.32.	1	0	0	1	1	0	1	0	0					
101	No	2	0	0	14	3	2	3	f	2.8.18.32.	1	0	0	1	1	0	1	0	0					
102	Lr	2	1	0	14	3	3	3	d	2.8.18.32.	1	0	0	1	1	0	1	0	0					

103	Rf	104	7	4	267	16	0	0	1.57	0	0	0	0	23	0	0	0	0	579
104	Db	105	7	5	268	12	0	0	1.49	0	0	0	0	58	0	0	0	0	0
105	Sg	106	7	6	269	12	0	0	1.43	0	0	0	0	19	0	0	0	0	0
106	Bh	107	7	7	270	10	0	0	1.41	0	0	0	0	0	0	0	0	0	0
107	Hs	108	7	8	269	10	0	0	1.34	0	0	0	0	0	0	0	0	0	0
108	Mt	109	7	9	278	10	0	0	1.29	0	0	0	0	0	0	0	0	0	0
109	Ds	110	7	10	281	10	0	0	1.28	0	0	0	0	0	0	0	0	0	0
110	Rg	111	7	11	280	12	0	0	1.21	0	0	0	0	0	0	0	0	0	0
111	Cn	112	7	12	285	17	0	0	1.22	0	0	0	0	0	0	0	0	0	0
112	Nh	113	7	13	286	18	0	0	1.36	0	0	0	0	0	0	0	0	0	0
113	Fl	114	7	14	289	21	0	0	1.43	0	0	0	0	0	0	0	0	0	0
114	Mc	115	7	15	289	22	0	0	1.62	0	0	0	0	0	0	0	0	0	0
115	Lv	116	7	16	293	26	0	0	1.75	0	0	0	0	0	0	0	0	0	0
116	Ts	117	7	17	294	41	0	0	1.65	0	0	0	0	0	0	0	0	0	0
117	Og	118	7	18	294	52	0	0	1.57	0	0	5.403	0	0.023	0	0	0	0	0

103	Rf	2	0	2	14	4	d	2.8.18.32.3	1	0	0	1	0	0	0	Gpa	GPa	GPa	K^-1
104	Db	2	0	3	14	5	d	2.8.18.32.3	1	0	0	1	0	0	0				
105	Sg	2	0	4	14	6	d	2.8.18.32.3	1	0	0	1	0	0	0				
106	Bh	2	0	5	14	7	d	2.8.18.32.3	1	0	0	1	0	0	0				
107	Hs	2	0	6	14		d	2.8.18.32.3	1	0	0	1	0	0	0				
108	Mt	2	0	7	14		d	2.8.18.32.32.15.2											
109	Ds	1	0	9	14		d	2.8.18.32.32.17.1											
110	Rg	1	0	10	14		d	2.8.18.32.32.18.1											
111	Cn	2	0	10	14		d	2.8.18.32.32.18.2											
112	Nh	2	1	10	14		p	2.8.18.32.32.18.3											
113	Fl	2	2	10	14		p	2.8.18.32.32.18.4											
114	Mc	2	3	10	14		p	2.8.18.32.32.18.5											
115	Lv	2	4	10	14		p	2.8.18.32.32.18.6											
116	Ts	2	5	10	14		p	2.8.18.32.32.18.7											
117	Og	2	6	10	14		p	2.8.18.32.32.18.8											
